# Evaluation of the feasibility, diagnostic yield, and clinical utility of rapid genome sequencing in infantile epilepsy (Gene-STEPS): an international, multicentre, pilot cohort study

**DOI:** 10.1016/S1474-4422(23)00246-6

**Published:** 2023-09

**Authors:** Alissa M D'Gama, Sarah Mulhern, Beth R Sheidley, Fadil Boodhoo, Sarah Buts, Natalie J Chandler, Joanna Cobb, Meredith Curtis, Edward J Higginbotham, Jonathon Holland, Tayyaba Khan, Julia Koh, Nicole S Y Liang, Lyndsey McRae, Sarah E Nesbitt, Brandon T Oby, Ben Paternoster, Alistair Patton, Graham Rose, Elizabeth Scotchman, Rozalia Valentine, Kimberly N Wiltrout, Gene-STEPS Study Group, IPCHiP Executive Committee, Robin Z Hayeems, Puneet Jain, Sebastian Lunke, Christian R Marshall, Shira Rockowitz, Neil J Sebire, Zornitza Stark, Susan M White, Lyn S Chitty, J Helen Cross, Ingrid E Scheffer, Vann Chau, Gregory Costain, Annapurna Poduri, Katherine B Howell, Amy McTague

**Affiliations:** a Epilepsy Genetics Program, Division of Epilepsy and Neurophysiology, Department of Neurology, Boston Children's Hospital, Boston, MA, USA; b Division of Newborn Medicine, Department of Pediatrics, Boston Children's Hospital, Boston, MA, USA; c Department of Pediatrics, Harvard Medical School, Boston, MA, USA; d Department of Neurology, Harvard Medical School, Boston, MA, USA; e Victorian Clinical Genetics Service, Melbourne, VIC, Australia; f Murdoch Children's Research Institute, Melbourne, VIC, Australia; g Department of Neurology, Great Ormond Street Hospital, London, UK; h Department of Paediatric Neurology, Aachen University Hospital, Germany; i North Thames Genomic Laboratory Hub, Great Ormond Street NHS Foundation Trust, London, UK; j Division of Genome Diagnostics, Hospital for Sick Children, Toronto, ON, Canada; k Program in Genetics and Genome Biology, SickKids Research Institute, Toronto, ON, Canada; l Program in Child Health Evaluative Sciences, SickKids Research Institute, Toronto, ON, Canada; m Department of Genetic Counselling, Hospital for Sick Children, Toronto, ON, Canada; n Department of Molecular Genetics, University of Toronto, Toronto, ON, Canada; o Department of Laboratory Medicine and Pathobiology, University of Toronto, Toronto, ON, Canada; p Division of Neurology, Department of Paediatrics, Hospital for Sick Children, Toronto, ON, Canada; q Genetics and Genomic Medicine, UCL Great Ormond Street Institute of Child Health, London, UK; r Developmental Neurosciences, UCL Great Ormond Street Institute of Child Health, London, UK; s Department of Paediatrics, Frimley Park Hospital, Frimley Health NHS Foundation Trust, Frimley, UK; t Institute of Health Policy, Management, and Evaluation, University of Toronto, Toronto, ON, Canada; u Department of Paediatrics, Temerty Faculty of Medicine, University of Toronto, Toronto, ON, Canada; v Department of Pathology, University of Melbourne, Melbourne, VIC, Australia; w Department of Paediatrics, University of Melbourne, Melbourne, VIC, Australia; x Department of Medicine, University of Melbourne, Melbourne, VIC, Australia; y The Manton Center for Orphan Disease Research, Division of Genetics and Genomics, Boston Children's Hospital, Boston, MA, USA; z Research Computing, Boston Children's Hospital, Boston, MA, USA; aa Division of Genetics and Genomics, Boston Children's Hospital, Boston, MA, USA; ab DRIVE Centre, Great Ormond Street Hospital for Children, London, UK; ac Austin Health, and Florey Institute of Neuroscience and Mental Health, Melbourne, VIC, Australia; ad Department of Neurology, Royal Children's Hospital, Melbourne, VIC, Australia; ae Division of Clinical and Metabolic Genetics, Department of Paediatrics, Hospital for Sick Children, Toronto, ON, Canada; af Developmental Neurosciences, Zayed Centre for Research into Rare Disease in Children, UCL Great Ormond Street Institute of Child Health, London, UK

## Abstract

**Background:**

Most neonatal and infantile-onset epilepsies have presumed genetic aetiologies, and early genetic diagnoses have the potential to inform clinical management and improve outcomes. We therefore aimed to determine the feasibility, diagnostic yield, and clinical utility of rapid genome sequencing in this population.

**Methods:**

We conducted an international, multicentre, cohort study (Gene-STEPS), which is a pilot study of the International Precision Child Health Partnership (IPCHiP). IPCHiP is a consortium of four paediatric centres with tertiary-level subspecialty services in Australia, Canada, the UK, and the USA. We recruited infants with new-onset epilepsy or complex febrile seizures from IPCHiP centres, who were younger than 12 months at seizure onset. We excluded infants with simple febrile seizures, acute provoked seizures, known acquired cause, or known genetic cause. Blood samples were collected from probands and available biological parents. Clinical data were collected from medical records, treating clinicians, and parents. Trio genome sequencing was done when both parents were available, and duo or singleton genome sequencing was done when one or neither parent was available. Site-specific protocols were used for DNA extraction and library preparation. Rapid genome sequencing and analysis was done at clinically accredited laboratories, and results were returned to families. We analysed summary statistics for cohort demographic and clinical characteristics and the timing, diagnostic yield, and clinical impact of rapid genome sequencing.

**Findings:**

Between Sept 1, 2021, and Aug 31, 2022, we enrolled 100 infants with new-onset epilepsy, of whom 41 (41%) were girls and 59 (59%) were boys. Median age of seizure onset was 128 days (IQR 46–192). For 43 (43% [binomial distribution 95% CI 33–53]) of 100 infants, we identified genetic diagnoses, with a median time from seizure onset to rapid genome sequencing result of 37 days (IQR 25–59). Genetic diagnosis was associated with neonatal seizure onset versus infantile seizure onset (14 [74%] of 19 *vs* 29 [36%] of 81; p=0·0027), referral setting (12 [71%] of 17 for intensive care, 19 [44%] of 43 non-intensive care inpatient, and 12 [30%] of 40 outpatient; p=0·0178), and epilepsy syndrome (13 [87%] of 15 for self-limited epilepsies, 18 [35%] of 51 for developmental and epileptic encephalopathies, 12 [35%] of 34 for other syndromes; p=0·001). Rapid genome sequencing revealed genetic heterogeneity, with 34 unique genes or genomic regions implicated. Genetic diagnoses had immediate clinical utility, informing treatment (24 [56%] of 43), additional evaluation (28 [65%]), prognosis (37 [86%]), and recurrence risk counselling (all cases).

**Interpretation:**

Our findings support the feasibility of implementation of rapid genome sequencing in the clinical care of infants with new-onset epilepsy. Longitudinal follow-up is needed to further assess the role of rapid genetic diagnosis in improving clinical, quality-of-life, and economic outcomes.

**Funding:**

American Academy of Pediatrics, Boston Children's Hospital Children's Rare Disease Cohorts Initiative, Canadian Institutes of Health Research, Epilepsy Canada, Feiga Bresver Academic Foundation, Great Ormond Street Hospital Charity, Medical Research Council, Murdoch Children's Research Institute, National Institute of Child Health and Human Development, National Institute for Health and Care Research Great Ormond Street Hospital Biomedical Research Centre, One8 Foundation, Ontario Brain Institute, Robinson Family Initiative for Transformational Research, The Royal Children's Hospital Foundation, University of Toronto McLaughlin Centre.

**Research in context**

**Evidence before this study**

We searched PubMed using the terms “epilepsy” OR “seizure(s)” AND “rapid” AND “sequencing” for studies published from database inception to Jan 1, 2023, with no language restrictions. We identified case reports of rapid exome or genome sequencing in patients with epilepsy and several studies of rapid exome or genome sequencing in critically ill paediatric cohorts recruited from neonatal and paediatric intensive care units, including some participants with seizures. We also identified a recent systematic review of genetic testing in the epilepsies, which found the highest diagnostic yield for (non-rapid) genome sequencing (48%) followed by exome sequencing (24%). No studies of rapid exome or genome sequencing (ie, with results available within weeks) in epilepsy cohorts exist.

**Added value of this study**

We report an international, multicentre, cohort study of the feasibility, diagnostic yield, and clinical utility of rapid genome sequencing in 100 infants with new-onset epilepsy, using trio-based analyses when parental DNA was available. To date, this study is the first to evaluate rapid sequencing in a disease-specific cohort and the first study consisting of patients mostly outside an intensive care setting. First, we show that rapid genome sequencing has high diagnostic yield (43 [43%] of 100 infants) in infantile epilepsy and demonstrate the feasibility of rapid turnaround for participants recruited from intensive care, non-intensive care inpatient, and outpatient settings across multiple health-care systems. Second, we demonstrate marked genetic heterogeneity across our cohort and demonstrate the ability of rapid genome sequencing to identify genetic diagnoses missed by standard-of-care genetic testing. Third, we observed that most parents of infants with newly diagnosed epilepsy are interested in rapid sequencing, and we demonstrate immediate clinical utility of genetic diagnoses for infants and their families in most cases.

**Implications of all the available evidence**

The findings from this study strongly support the implementation of rapid genome sequencing in the clinical evaluation of infants with new-onset epilepsy. These findings also enhance our understanding of underlying genetic mechanisms of epilepsy. Future research will be needed to understand the personal and long-term utility of early genetic diagnosis in infantile epilepsy. This study provides a framework for advancing precision health that can be implemented for other unexplained conditions beyond epilepsy.

## Introduction

Infantile-onset epilepsies range in severity from self-limited epilepsies to the larger group of developmental and epileptic encephalopathies.^1^ The incidence of infantile-onset epilepsies is one in 1200. Patients with developmental and epileptic encephalopathies have drug-resistant seizures, severe developmental impairment, and high mortality risk, with important psychosocial implications for families and substantial economic costs for health systems.^1^, ^2^

Infantile-onset epilepsies often have genetic aetiologies (>800 genes implicated).^3^ Numerous studies, including a systematic review,^4^ show high diagnostic yield and cost-effectiveness of gene panels and exome sequencing in early-onset epilepsies, with genetic testing now considered a first-line investigation.^5^, ^6^, ^7^, ^8^ Genome sequencing further increases diagnostic yield,^4^ but has not been studied in unselected infantile epilepsy cohorts. In rare disease, genome sequencing, especially trio genome sequencing, has demonstrated substantial diagnostic yield.^9^

For infants with epilepsy, the identification of a precise diagnosis can guide clinical management and inform prognosis regarding seizure control, developmental outcome, and potential comorbidities. A growing number of genetic epilepsies have precision treatment implications, including four common infantile epilepsy genes (*KCNQ2*, *PRRT2*, *SCN1A*, *SLC2A1*).^7^ Although genetic therapies are not currently available for most epilepsies, tailoring of antiseizure medication is often possible.^10^ Furthermore, genetic diagnoses could inform eligibility for clinical trials or non-antiseizure medication treatment (eg, epilepsy surgery) and enable precise genetic counselling. In a few studies of the effect of non-genome sequencing genetic testing in epilepsy, genetic diagnoses affected management in 36–72% of cases.^11^, ^12^, ^13^, ^14^, ^15^

Although rapid genetic testing and prompt implementation of individualised treatment, where available, will possibly improve outcomes, a major challenge is that testing often takes months to years, with infants having progressive neurological sequelae from uncontrolled seizures or underlying disease.^16^ Studies done in neonatal intensive care units (NICUs) and paediatric intensive care units (PICUs) demonstrate high diagnostic yield of rapid (ie, weeks) and ultrarapid (ie, days) genome sequencing for a range of conditions, with clinical utility and reduction in health-care costs.^17^, ^18^, ^19^ To date, rapid genome sequencing has been undertaken primarily in ICUs, and the effect of prompt genetic diagnoses in infants with epilepsy has not been established. In this study, we therefore aimed to demonstrate the feasibility of rapid genome sequencing and investigate the diagnostic yield and clinical utility for infants with new-onset epilepsy.

## Methods

### Study design and cohort

We conducted an international, multicentre, cohort study (Gene-Shortening Time of Evaluation in Paediatric epilepsy Services [STEPS]), which is a pilot study of the International Precision Child Health Partnership (IPCHiP). This partnership is a consortium of four paediatric centres with tertiary-level subspecialty services, created to advance precision child health: Melbourne Children's Campus (MCC; Murdoch Children's Research Institute and The Royal Children's Hospital) in Australia; The Hospital for Sick Children (SickKids) in Canada; University College London Great Ormond Street Institute of Child Health (UCL GOS ICH) in the UK; and Boston Children's Hospital (BCH) in the USA.

We recruited infants with new-onset epilepsy or complex febrile seizures from the IPCHiP centres. Potentially eligible infants were identified by the study team and treating clinicians. The study team reviewed medical records and determined eligibility in discussion with treating clinicians. Infants younger than 12 months at seizure onset and recruited within 6 weeks of study site presentation were enrolled into the study with parental consent. We excluded infants with simple febrile seizures, acute provoked seizures, known acquired cause, or known genetic cause (ie, diagnostic genetic test result or clinical findings consistent with a monogenic syndrome, such as tuberous sclerosis complex). Brain MRI was reviewed to confirm lack of acquired aetiology at screening or as soon as available. We did not exclude infants with structural brain malformations without known genetic cause, or infants with a previous non-diagnostic or concurrent in-progress genetic testing, so as not to disrupt site-specific clinical standard of care. We worked with certified interpreters at each site for non-English-speaking families.

This study was approved by each site's institutional review boards and human ethics research committees. We obtained written informed consent from parents for research enrolment, clinically accredited rapid genome sequencing, and results return.

### Clinical data

Clinical data were collected from medical records, treating clinicians, and parents. We documented study site, referral setting (outpatient, non-intensive care inpatient, NICU, PICU), sex, parent-reported race, gestational age, family medical history, epilepsy details (age at seizure onset, seizure type, EEG findings), development before seizure onset, developmental plateau or regression following seizure onset, other neurological and non-neurological features, MRI findings, previous and concurrent genetic testing, and, if applicable, age at death. We classified epilepsy syndrome using the International League Against Epilepsy definitions, and we classified an epilepsy syndrome as other when the participant's presentation did not fit diagnostic criteria for one of those definitions.^1^

### Rapid genome sequencing

Blood samples were collected from probands and available biological parents. We did trio genome sequencing when both parents were available, and duo or singleton genome sequencing when one or neither parent was available. Site-specific protocols were used for DNA extraction, library preparation, genome sequencing, variant identification, and validation at clinically accredited laboratories (appendix pp 2–3). All sites performed genome-wide analysis for single nucleotide variants, small insertions and deletions, and copy number variants; the laboratory used by BCH was also clinically accredited to report short tandem repeat expansions in *FMR1* and *DMPK*. Variant classification used standardised criteria (American College of Medical Genetics and Genomics^20^ or Association for Clinical Genomic Science). Site-specific policies were followed for reporting variants of uncertain significance and secondary or incidental findings (appendix pp 2–3). Infants with pathogenic or likely pathogenic variants in genes consistent with phenotypes and modes of inheritance were considered to have diagnostic rapid genome sequencing. For infants with variants of uncertain significance that were plausibly explanatory (ie, no data ruled out pathogenicity, but insufficient data were present to classify as pathogenic or likely pathogenic variants), we reviewed medical records for clinical features or further investigations to support pathogenicity to deem variants clinically diagnostic.

### Effect of rapid genome sequencing

We documented age at study site presentation, enrolment, blood collection, and rapid genome sequencing result. Short-term clinical utility (ie, to December, 2022) of rapid genome sequencing was assessed through medical records and treating clinicians. We defined clinical utility as actual influence on treatment, potential for precision therapy, additional investigation indicated or avoided, additional prognostic information, influence on goals of care, or influence on genetic counselling (beyond recurrence risk).

### Statistical analysis

We analysed summary statistics for cohort demographic and clinical characteristics and the timing, diagnostic yield, and clinical effect of rapid genome sequencing. We analysed associations of demographic features, clinical features, and timing with diagnostic rapid genome sequencing using a two-tailed χ^2^ test, Fisher's exact test, Mann-Whitney test, or Kruskal-Wallis test (based on normality assessment using Kolmogorov-Smirnov and Shapiro-Wilk tests) using the program SPSS (version 27.0), with statistical significance set at p<0·05.

### Role of the funding source

The funders of the study had no role in study design, data collection, data analysis, data interpretation, writing of the report, or the decision to submit for publication.

## Results

**Figure 1:**
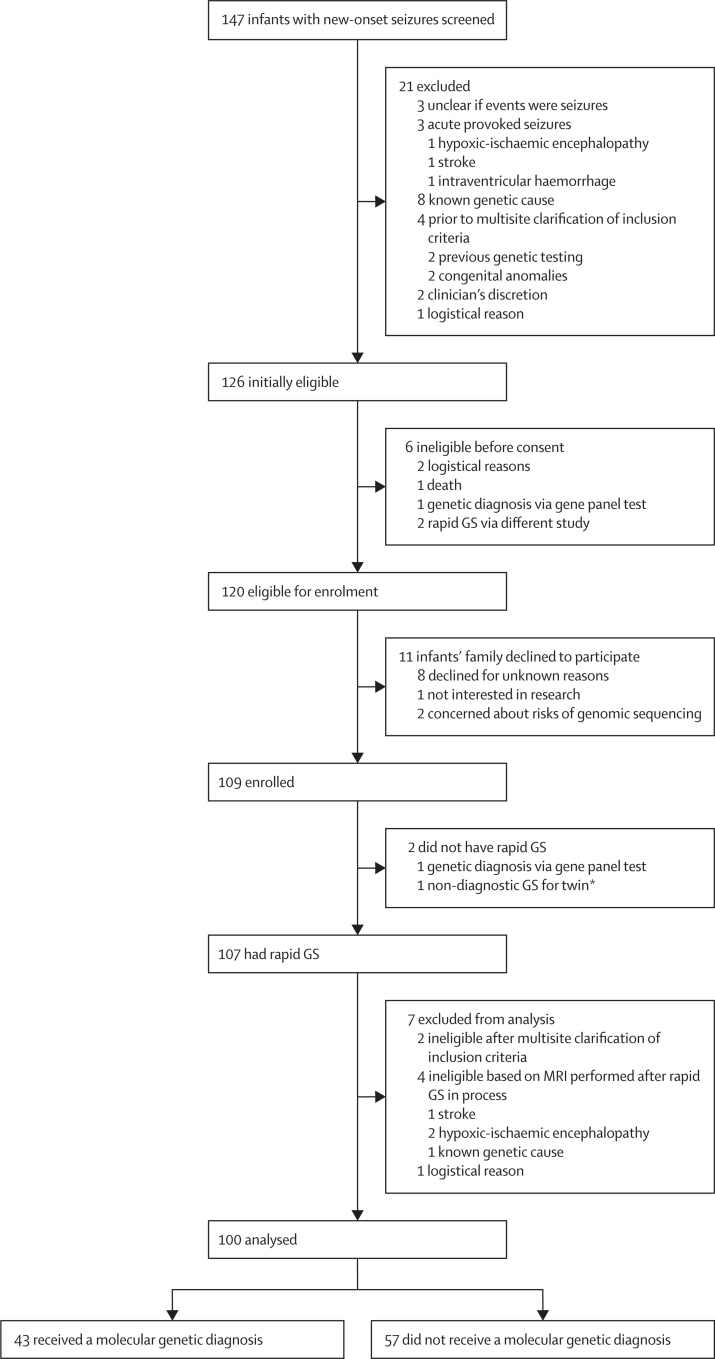
Study profile GS=genome sequencing. *Not via this study.

Between Sept 1, 2021, and Aug 31, 2022, we screened 147 infants with seizures and confirmed 120 (82%) as eligible for enrolment (figure 1). Parents of 109 (91%) of 120 eligible infants consented; two (2%) infants became ineligible after consent and seven (6%) after rapid genome sequencing commenced (eg, MRI showed evidence of stroke). For the remaining cohort of 100 infants (59 [59%] were boys and 41 [41%] were girls), 34 (34%) were enrolled from BCH and 22 (22%) each from MCC, SickKids, and UCL GOS ICH. As reported by parents, 63 (63%) of 100 infants were White, 18 (18%) were Asian, eight (8%) were of multiple races, six (6%) were Black, three (3%) were Middle Eastern, and two (2%) were reported as other. 60 (60%) of 100 infants were recruited from inpatient settings (13 [13%] NICU, four [4%] PICU, and 43 [43%] non-intensive care inpatient) and 40 (40%) from outpatient settings (appendix pp 5–8).

Median age at seizure onset was 128 days (IQR 46–192), with neonatal seizure onset (<44 weeks postmenstrual age) occurring in 19 (19%) of 100 infants (table 1). Focal seizures were the initial seizure type in 50 (50%) of 100 infants. 51 (51%) of 100 infants had developmental and epileptic encephalopathies—the most common was infantile epileptic spasms syndrome (32 [32%] of 100) followed by early infantile developmental and epileptic encephalopathy (13 [13%])—15 (15%) had self-limited epilepsies, and 34 (34%) had other syndromes.^1^ MRI revealed malformations of cortical development in 11 (11%) of 100 infants. Of the 81 infants with infantile-onset seizures (between 44 weeks postmenstrual age and 12 months), 20 (25%) had developmental delay before seizure onset and 25 (31%) had developmental plateau or regression following seizure onset (appendix pp 9–13).

**Table 1:** Participant demographics and clinical presentation

		**Total (n=100)**	**Genetic diagnosis (n=43)**	**No genetic diagnosis (n=57)**	p value*
Sex		..	..	..	0·57
	Male	59 (59%)	24/59 (41%)	35/59 (59%)	..
	Female	41 (41%)	19/41 (46%)	22/41 (54%)	..
Prematurity (<37 weeks gestational age)		15 (15%)	6/15 (40%)	9/15 (60%)	0·80
Age at seizure onset (days)		128 (46–192)	105 (17–151)	153 (78–200)	0·0163
Neonatal seizure onset (<44 weeks postmenstrual age)		19 (19%)	14/19 (74%)	5/19 (26%)	0·0027
Seizure onset to site presentation (days)		7 (1–24)	2 (0–15)	13 (3–27)	0·0164
Referral setting		..	..	..	0·0178†
	Intensive care	17 (17%)	12/17 (71%)	5/17 (29%)	..
	Non-intensive care inpatient	43 (43%)	19/43 (44%)	24/43 (56%)	..
	Outpatient	40 (40%)	12/40 (30%)	28/40 (70%)	..
Deceased in first year of life		6 (6%)	4/6 (67%)	2/6 (33%)	0·40
Seizure type at onset		..	..	..	0·29‡
	Focal	50 (50%)	25/50 (50%)	25/50 (50%)	..
	Generalised	35 (35%)	12/35 (34%)	23/35 (66%)	..
	Both	7 (7%)	4/7 (57%)	3/7 (43%)	..
	Unknown	8 (8%)	2/8 (25%)	6/8 (75%)	..
Epilepsy syndrome at onset		..	..	..	0·001§
Self-limited epilepsies		15 (15%)	13/15 (87%)	2/15 (13%)	..
	Self-limited neonatal epilepsy	3/15 (20%)	3/3 (100%)	0	..
	Self-limited infantile epilepsy	11/15 (73%)	9/11 (82%)	2/11 (18%)	..
	Self-limited familial neonatal-infantile epilepsy	1/15 (7%)	1/1 (100%)	0	..
DEEs		51 (51%)	18/51 (35%)	33/51 (65%)	..
	Early infantile DEE	13/51 (25%)	7/13 (54%)	6/13 (46%)	..
	Infantile epileptic spasms syndrome	32/51 (63%)	6/32 (19%)	26/32 (81%)	..
	Dravet syndrome	2/51 (4%)	2/2 (100%)	0	..
	Other DEEs	4/51 (8%)	3/4 (75%)	1/4 (25%)	..
Other		34 (34%)	12/34 (35%)	22/34 (65%)	..
	Other focal epilepsy	24/34 (71%)	7/24 (29%)	17/24 (71%)	..
	Complex febrile seizures	3/34 (9%)	1/3 (33%)	2/3 (67%)	..
	Other syndrome	7/34 (21%)	4/7 (57%)	3/7 (43%)	..
Other clinical features at onset					
	Developmental delay before onset for infantile-onset cases	20/81 (25%)	11/20 (55%)	9/20 (45%)	0·0391
	Developmental regression following onset for infantile-onset cases	25/81 (31%)	9/25 (36%)	16/25 (64%)	0·98
	Malformation of cortical development	11 (11%)	6/11 (55%)	5/11 (45%)	0·52
	Abnormal tone (hypotonia, hypertonia, or dystonia)	27 (27%)	15/27 (56%)	12/27 (44%)	0·12
	Abnormal head size (macrocephaly or microcephaly)	8 (8%)	7/8 (88%)	1/8 (12%)	0·0195
	Dysmorphic features	8 (8%)	5/8 (63%)	3/8 (37%)	0·28
	Family history of seizures (first-degree or second-degree relative)	29 (29%)	12/29 (41%)	17/29 (59%)	0·83
	Parental consanguinity	6 (6%)	3/6 (50%)	3/6 (50%)	>0·99
Study enrolment to genome sequencing result (days)		21 (15–23)	20 (15–25)	21 (15–22)	0·90
Age at genome sequencing result (days)		172 (91–250)	140 (60–231)	204 (126–265)	0·0245

Data are n (%), n/N (%), or median (IQR), unless otherwise specified. DEE=developmental and epileptic encephalopathy.

* Uncorrected p value calculated using two-tailed χ^2^, Fisher's exact, or Mann-Whitney test, as appropriate.

† Comparing genetic diagnosis versus no genetic diagnosis across the three categories of referral source.

‡ Comparing genetic diagnosis versus no genetic diagnosis across the four categories of seizure type at onset.

§ Comparing genetic diagnosis versus no genetic diagnosis across the three main categories of epilepsy syndrome at onset (self-limited epilepsies, DEEs, and other).

**Figure 2:**
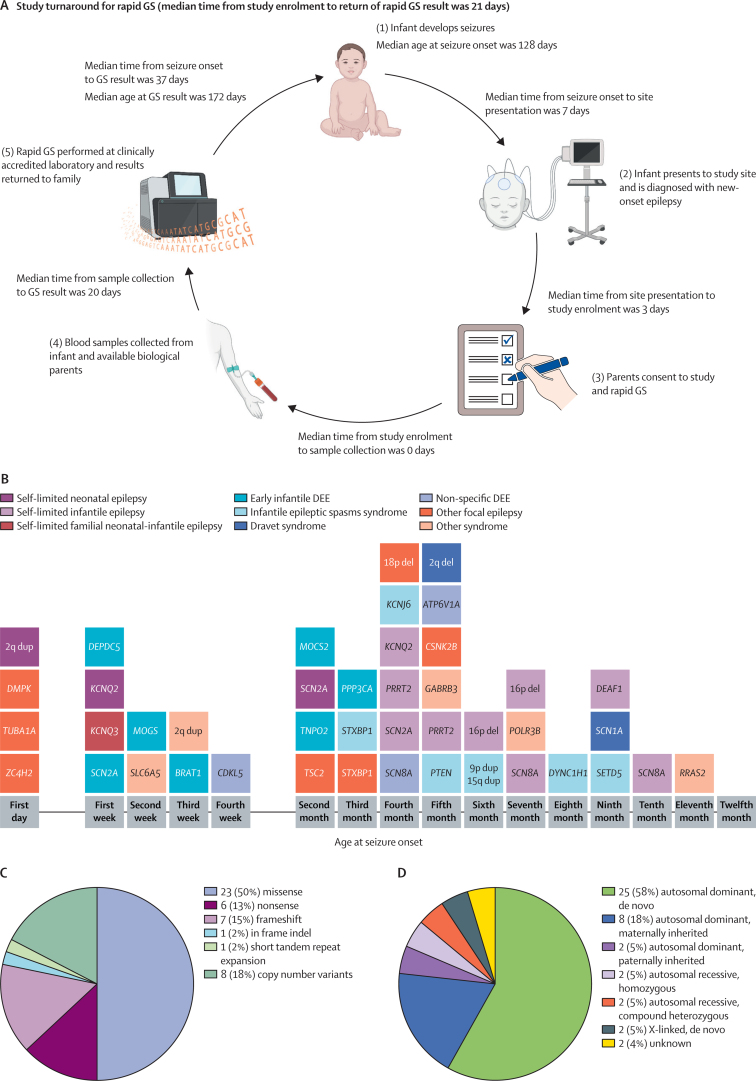
Rapid GS workflow and summary of genetic diagnoses (A) Rapid GS workflow and time intervals, created with BioRender.com. (B) Genetic diagnoses arranged by age at seizure onset. Each square represents an infant who received a genetic diagnosis. The affected gene or genomic region is denoted in the square. The infant with a diagnostic *SCN2A* variant and seizure onset in the second month of life classified as self-limited neonatal epilepsy was born prematurely and was younger than 44 weeks postmenstrual age at seizure onset. (C) Types of variants in diagnostic cases. Data are n (%) of 46 total variants. (D) Mode of inheritance of variants in diagnostic cases. Data are n (%) of 43 total diagnoses. GS=genome sequencing. DEE=developmental and epileptic encephalopathy.

Median time from seizure onset to site presentation was 7 days (IQR 1–24), from site presentation to enrolment was 3 days (1–9), from enrolment to proband sample collection was 0 days (0–1), and from sample collection to rapid genome sequencing result was 20 days (14–22; figure 2A). 91 (91%) of 100 families had trio genome sequencing, eight (8%) had duo genome sequencing, and one (1%) had singleton genome sequencing. Median study turnaround time from enrolment to rapid genome sequencing result was 21 days (IQR 15–23), shorter at one site (BCH) than the others (median 15 days *vs* 21–25 days; adjusted p<0·05 for pairwise comparisons) and not significantly different between referral settings. Median time from seizure onset to rapid genome sequencing result was 37 days (IQR 25–59) and median age at rapid genome sequencing result was 172 days (91–250), following median age at seizure onset of 128 days (appendix pp 4, 14–17).

We identified genetic diagnoses for 43 (43% [binomial distribution 95% CI 33–53]) of 100 infants with new-onset epilepsy (table 2), with similar yield across sites (41–45%) and varied yield by referral setting: 12 (71%) of 17 for intensive care, 19 (44%) of 43 non-intensive care inpatient, and 12 (30%) of 40 outpatient (p=0·0178). 39 (91%) of 43 infants had pathogenic or likely pathogenic variants and four (9%) had variants of uncertain significance considered clinically diagnostic. Infants with diagnostic rapid genome sequencing were younger at seizure onset than were infants with non-diagnostic rapid genome sequencing (median 105 days *vs* 153 days; p=0·0163). Diagnostic yield was higher in infants with neonatal-onset seizures versus infantile-onset seizures (14 [74%] of 19 *vs* 29 [36%] of 81; p=0·0027), with previous developmental delay versus without previous developmental delay (11 [55%] of 20 *vs* 18 [30%] of 61; p=0·0391), and with abnormal head size versus normocephaly (seven [88%] of eight *vs* 36 [39%] of 92; p=0·0195). Diagnostic yield varied by epilepsy syndrome: 13 (87%) of 15 for self-limited epilepsy; 18 (35%) of 51 for developmental and epileptic encephalopathies, including seven (54%) of 13 with early infantile developmental and epileptic encephalopathy and six (19%) of 32 with infantile epileptic spasms syndrome; and 12 (35%) of 34 for other syndromes, including 11 (35%) of 31 with unclassified epilepsy and one (33%) of three with complex febrile seizures (p=0·001; table 1).

**Table 2:** Genetic diagnoses made by rapid genome sequencing

	**Sex**	**Referral source**	**Age at onset (days)**	**Age at results (days)**	**Epilepsy syndrome**	**Developmental delay**	**Developmental regression**	**Other clinical features**	**Gene or cytoband**	**Variant**	**Zygosity**	**Inheritance**	**Classifi-cation**	**Other genetic testing (non-diagnostic unless otherwise noted)**
001	Male	NICU	4	26	EIDEE	NA	NA	Unilateral PMG	*DEPDC5*	c.3061del, p.I1021Ffs*58	Het	Maternal	P	Previous CMA
002	Male	Inpatient	224	241	IESS	Yes	No	Hypotonia	*DYNC1H1*	c.6989G>A, p.G2330E	Het	De novo	LP	Concurrent CMA, panel
011	Male	Inpatient	42	120	EIDEE	Yes	No	MCD	*TNPO2*	c.466G>A, p.D156N	Het	De novo	P	..
014	Female	Inpatient	64	81	EIDEE	No	No	..	*PPP3CA*	c.1255_1256del, p.S419Cfs*31	Het	De novo	P	Concurrent CMA
015	Male	Inpatient	149	161	IESS	No	No	MCD	*PTEN*	c.49C>T, p.Q17*	Het	De novo	P	..
016	Male	NICU	1	37	Focal	NA	NA	Premature (33 weeks), hypotonia, AMC	*DMPK*	c.*224_226CTG[>200]	Het	Maternal*	P	Previous CMA, rES, mito
018	Female	Inpatient	123	155	DS-like	Yes	No	Hypotonia	2q24.2q24.3	Deletion	Het	De novo	P	..
021	Female	Outpatient	291	357	SeLIE	No	No	..	*SCN8A*	c.3926G>A, p.R1309Q	Het	De novo	P	..
026	Male	Inpatient	251	271	IESS	Yes	Yes	Hypotonia, DF	*SETD5*	c.1967T>G, p.L656*	Het	De novo	P	..
027	Female	NICU	1	20	Focal	NA	NA	Hypertonia, DF	*ZC4H2*	c.450del, p.I151Sfs*36	Het	De novo	P	..
028	Female	NICU	35	58	SeLNE	NA	NA	Premature (33 weeks)	*SCN2A*	c.662T>C, p.V221A	Het	De novo	LP	..
029	Male	NICU	10	28	EIDEE	NA	NA	Callosal dysgenesis, hypotonia, DF, PRS	*MOGS*	c.[1461G>C];[2470G>A], p.[E487D];[G824S]	Comp het	Maternal; paternal	LP; VUS†	Previous prenatal karyotype, CMA
030	Male	PICU	48	73	EIDEE	NA	NA	Premature (36 weeks), DF	*MOCS2*	c.226G>A, p.G76R	Hom	Both	P	Previous CMA
032	Female	Inpatient	126	155	Focal	No	No	..	*CSNK2B*	c.557G>A, p.R186K	Het	De novo	LP	..
034	Female	NICU	115	139	SeLIe	No	No	..	*SCN2A*	c.5287T>A, p.S1763T	Het	Paternal*	VUS‡	..
035	Male	Outpatient	66	144	Focal	No	No	..	*STXBP1*	c.260T>C, p.L87P	Het	De novo	LP	Concurrent CMA, panel (identified)
037	Male	NICU	18	61	EIDEE	NA	NA	Premature (31 weeks), hypotonia, DF	*BRAT1*	c.[1593G>A];[294dup], p.[W531*];[L99Tfs*92]	Comp het	Maternal; paternal	P; P	Concurrent CMA
039	Male	Outpatient	121	386	Other	No	Yes	..	*GABRB3*	c.238A>G, p.M80V	Het	De novo	LP	Concurrent CMA, panel (identified)
041	Male	Inpatient	99	130	SeLIE	No	No	..	*KCNQ2*	c.998G>A, p.R333Q	Het	De novo	P	Concurrent panel (identified)
045	Male	Outpatient	206	266	Other	No	Yes	Axial hypotonia	*POLR3B*	c.1765G>A, p.G589R	Het	De novo	VUS§	Concurrent panel
046	Female	Outpatient	16	47	Other	NA	NA	..	2q24.3q32.1	Duplication	Het	Mosaic	P	Concurrent CMA (identified), panel (identified)
050	Female	Inpatient	138	160	DEE	Yes	Yes	Dystonia, OA	*ATP6V1A*	c.761G>A, p.C254Y	Het	De novo	LP	Concurrent CMA, ES (identified)
052	Female	Outpatient	27	52	DEE	No	Yes	..	*CDKL5*	c.1648C>T, p.R550*	Het	De novo	P	Concurrent CMA, panel (identified)
054	Male	Outpatient	86	128	IESS	Yes	Yes	Premature (36 weeks), dyskinesia	*STXBP1*	c.703C>T, p.R235*	Het	De novo	P	Concurrent CMA, panel (identified)
056	Female	Outpatient	108	167	DEE	No	Yes	**..**	*SCN8A*	c.5615G>A, p.R1872Q	Het	De novo	P	Concurrent CMA, panel (identified)
058	Male	Inpatient	53	110	Focal	No	No	MCD	*TSC2*	c.3598C>T, p.R1200W	Het	Maternal*	P	..
060	Male	Inpatient	146	169	SeLIE	No	No	..	*PRRT2*	c.649dup, p.R217Pfs*8	Het	Maternal¶	P	..
061	Female	Inpatient	114	140	Focal	Yes	No	..	18p11.32p11.21	Deletion	Het	De novo	P	Concurrent CMA (identified)
065	Female	Inpatient	0	53	SeLNE	NA	NA	VSD	2q24.1q24.3	Duplication	Het	Unknown	P	..
067	Female	Outpatient	326	362	CFS	Yes	No	Hypotonia	*RRAS2*	c.68G>A, p.G23D	Het	De novo	P	Previous CMA and fragile X
073	Male	Inpatient	209	235	SeLIE	No	No	..	16p11.2	Deletion	Het	Not maternal	P	..
074	Male	PICU	251	287	DS	No	No	..	*SCN1A*	c.4867G>C, p.E1623Q	Het	Maternal*	LP	..
076	Male	Inpatient	1	99	Focal	NA	NA	MCD, abnormal tone, movement disorder, microcephaly	*TUBA1A*	c.168_171delinsAGCATTCAGAGAT,p.G57delinsAFRD	Het	De novo	LP	..
078	Male	Inpatient	163	227	IESS	Yes	Yes	Hypotonia	9pterq22.33; 15q22.2qter	Duplication; duplication	Het	Mosaic	P	Concurrent CMA
082	Female	Outpatient	2	91	SeLNE	NA	NA	..	*KCNQ2*	c.1955del, p.P652Rfs*278	Het	Maternal	LP	Concurrent panel (identified)
084	Male	Inpatient	119	145	SeLIE	No	No	..	*PRRT2*	c.649C>T, p.R217*	Het	Maternal*	P	Concurrent CMA
086	Female	Inpatient	243	287	SeLIE	Yes	No	Hypotonia	*DEAF1*	c.709C>A, p.P237T	Het	De novo	LP	Concurrent CMA, panel
088	Female	Outpatient	105	275	IESS	Yes	Yes	..	*KCNJ6*	c.235G>A, p.D79N	Het	De novo	VUS‡	Concurrent CMA
091	Female	Outpatient	152	274	SeLIE	No	No	..	16p11.2	Deletion	Het	De novo	P	Concurrent CMA (identified), panel
092	Male	NICU	5	45	EIDEE	NA	NA	Hypoplastic corpus callosum, hypotonia, club feet, macrocephaly, ASD	*SCN2A*	c.415A>G, p.I139V	Het	Mosaic	LP	Concurrent CMA, panel (identified but classified as VUS)
093	Male	Inpatient	189	213	SeLIE	No	No	..	*SCN8A*	c.4441A>G, p.M1481V	Het	Maternal*	LP	..
099	Male	NICU	10	39	Other	NA	NA	Premature (32 weeks), abnormal tone	*SLC6A5*	c.296_297del, p.E99Gfs*22	Hom	Both	P	Concurrent panel
100	Female	NICU	4	24	SeLFNIE	NA	NA	..	*KCNQ3*	c.988C>T, p.R330C	Het	Paternal*	P	Concurrent panel (identified)

AMC=arthrogryposis multiplex congenita. ASD=atrial septal defect. CFS=complex febrile seizures. CMA=chromosomal microarray. Comp het=compound heterozygous. DF=dysmorphic features. DS=Dravet syndrome. EIDEE=early infantile developmental and epileptic encephalopathy. ES=exome sequencing. GA=gestational age. Het=heterozygous. Hom=homozygous. IESS=infantile epileptic spasms syndrome. LP=likely pathogenic. MCD=malformation of cortical development. Mito=mitochondrial sequencing. NA=not applicable. NICU=neonatal intensive care unit. OA=optic atrophy. P=pathogenic. PICU=paediatric intensive care unit. PMG=polymicrogyria. PRS=Pierre Robin sequence. rES=rapid exome sequencing. SeLFNIE=self-limited familial neonatal-infantile epilepsy. SeLIE=self-limited infantile epilepsy. SeLNE=self-limited neonatal epilepsy. VSD=ventricular septal defect. VUS=variant of uncertain significance.

* Parent from whom variant was inherited was also affected.

† Confirmed biochemically.

‡ Considered clinically diagnostic by clinical team.

§ Subsequently found to have abnormal nerve conduction testing.

¶ Parent from whom variant was inherited was suspected to also be affected.

The genetic diagnoses were heterogeneous, with only seven genes or chromosomal regions implicated more than once and 34 unique genes or genomic regions implicated (figure 2B). Of the 46 pathogenic variants, 37 (80%) were single nucleotide variants or small insertions or deletions, eight (18%) were copy number variants, and one (2%) was a short tandem repeat expansion (figure 2C). The most common inheritance mode was de novo autosomal dominant (ie, only one allele needed to be affected to cause disease; 25 [58%] of 43, including three cases with mosaic variants), followed by inherited autosomal dominant (ten [23%]; eight [80%] of ten parents were affected or suspected to be affected, of whom two [25%] learned they were affected through this study), autosomal recessive (four [9%]), and de novo X-linked (two [5%]); two (5%) were autosomal dominant with unknown inheritance (figure 2D).

In 15 cases, rapid genome sequencing identified genetic diagnoses not made by site-specific standard of care clinical testing (table 2): five (33%) with previous non-diagnostic testing and ten (67%) with concurrent non-diagnostic testing. In one infant, rapid genome sequencing detected a mosaic copy number variant (validated with karyotype) not identified on chromosomal microarray. In another infant, singleton gene panel identified a *SCN2A* variant classified as a variant of uncertain significance; trio rapid genome sequencing identified the variant as de novo, leading to the classification as likely pathogenic and facilitating immediate management changes.

Of the 57 infants with non-diagnostic genome sequencing, ten (18%) had variants of uncertain significance in genes potentially relevant to phenotypes (appendix pp 18–24). Secondary or incidental diagnostic findings were detected in five (5%) of 100 infants (appendix pp 25–26).

Clinical utility was present for 42 (98%) of 43 infants with genetic diagnoses (table 3; appendix pp 27–28). In one (2%) of 43 infants (case 099), the genetic diagnosis led to a new clinical diagnosis: an infant initially diagnosed with clinical seizures was also diagnosed with hyperekplexia after detection of a *SLC6A5* variant. Genetic diagnoses influenced treatment, predominantly antiseizure medication selection, in 24 (56%) of 43 infants; implicated potential precision therapies, regardless of whether used, in 21 (49%); and led to additional evaluation in 28 (65%; all had new subspecialty referrals and 11 [39%] of 28 new imaging or laboratory tests). Further evaluation was avoided for eight (19%) of 43 infants. In 37 (86%) of 43 infants, genetic diagnoses informed prognosis beyond that based on the epilepsy diagnosis (eg, likelihood of intellectual disability). For two (5%) of 43 infants (*MOGS*-congenital disorder of glycosylation and *BRAT1*-lethal neonatal rigidity and multifocal seizure syndrome), the genetic diagnoses supported decision making to redirect care to palliation. All families received genetic counselling, including recurrence risk counselling; 12 (28%) of 43 infants had genetic diagnoses that had health implications for parents or led to referral of additional family members for genetic testing. For non-diagnostic and secondary or incidental rapid genome sequencing results, clinical utility was present for 13 (23%) of 57 infants (appendix pp 27–28). In one infant (case 085), non-diagnostic rapid genome sequencing supported decision making to redirect care to palliation by helping to rule out potentially treatable aetiologies.

**Table 3:** Utility of genetic diagnoses

	**Gene**	**Any utility**	**Influence treatment**	**New workup**	**Avoid workup**	**Inform prognosis**	**Inform goals of care**	Inform genetic counselling*	**Potential precision therapy**†
Total (n=43)	..	42 (98%)	24 (56%)	28 (65%)	8 (19%)	37 (86%)	2 (5%)	12 (28%)	21 (49%)
001	*DEPDC5*	Yes	Yes	..	..	Yes	..	Yes	Yes
002	*DYNC1H1*	Yes	..	Yes	..	Yes	..	..	..
011	*TNPO2*	Yes	..	Yes	..	Yes	..	..	..
014	*PPP3CA*	Yes	..	Yes	..	Yes	..	..	..
015	*PTEN*	Yes	..	Yes	..	Yes	..	..	Yes
016	*DMPK*	Yes‡	..	..	..	..	..	Yes	..
018	2q del	Yes	Yes	..	..	Yes	..	..	Yes
021	*SCN8A*	Yes	Yes	..	..	Yes	..	Yes	Yes
026	*SETD5*	Yes	..	Yes	..	Yes	..	..	..
027	*ZC4H2*	Yes	..	Yes	..	Yes	..	..	..
028	*SCN2A*	Yes	Yes	..	..	Yes	..	..	Yes
029	*MOGS*	Yes	..	Yes	Yes	Yes	Yes	Yes	..
030	*MOCS2*	Yes	Yes	..	..	Yes	..	..	..
032	*CSNK2B*	Yes	..	..	..	Yes	..	..	..
034	*SCN2A*	Yes	Yes	..	..	Yes	..	..	Yes
035	*STXBP1*	Yes	Yes	Yes	Yes	Yes	..	..	..
037	*BRAT1*	Yes	..	Yes	Yes	Yes	Yes	..	..
039	*GABRB3*	Yes	..	Yes	Yes	..	..	..	..
041	*KCNQ2*	Yes	Yes	Yes	Yes	Yes	..	..	Yes
045	*POLR3B*	Yes	..	Yes	..	Yes	..	..	..
046	2q dup	Yes	Yes	Yes	Yes	Yes	..	..	Yes
050	*ATP6V1A*	Yes	..	Yes	..	Yes	..	..	..
052	*CDKL5*	Yes	Yes	Yes	..	Yes	..	..	..
054	*STXBP1*	Yes	Yes	Yes	..	Yes	..	..	..
056	*SCN8A*	Yes	Yes	Yes	Yes	Yes	..	..	Yes
058	*TSC2*	Yes	Yes	Yes	..	Yes	..	Yes	Yes
060	*PRRT2*	Yes	Yes	..	..	Yes	..	Yes	Yes
061	18p del	Yes	..	Yes	..	Yes	..	..	..
065	2q dup	Yes	Yes	..	..	Yes	..	..	Yes
067	*RRAS2*	Yes	..	Yes	..	..	..	Yes	..
073	16p del	Yes	Yes	Yes	..	Yes	..	..	Yes
074	*SCN1A*	Yes	Yes	Yes	..	Yes	..	Yes	Yes
076	*TUBA1A*	**..**	..	..	..	..	..	..	..
078	9p dup & 15q dup	Yes	..	Yes	..	..	..	..	..
082	*KCNQ2*	Yes	Yes	..	..	Yes	..	..	Yes
084	*PRRT2*	Yes	Yes	Yes	..	Yes	..	Yes	Yes
086	*DEAF1*	Yes	..	Yes	..	Yes	..	..	..
088	*KCNJ6*	Yes	..	Yes	..	..	..	..	..
091	16p del	Yes	Yes	Yes	..	Yes	..	..	Yes
092	*SCN2A*	Yes	Yes	..	..	Yes	..	..	Yes
093	*SCN8A*	Yes	Yes	..	..	Yes	..	Yes	Yes
099	*SLC6A5*	Yes	Yes	Yes	Yes	Yes	..	Yes	Yes
100	*KCNQ3*	Yes	Yes	..	..	Yes	..	Yes	Yes

Data are n (%), unless otherwise specified.

* All families received recurrence risk counselling based on the mode of inheritance of the diagnostic variants. Yes in this column refers to new health implication for parents or referral of additional family members for genetic testing for the diagnostic variants.

† Implication for precision treatment based on the genetic aetiology regardless of whether the treatment was used.

‡ Diagnosis did not have direct utility for this case as the infant died before the rapid genome sequencing result was available.

## Discussion

To the best of our knowledge, this international, multicentre Gene-STEPS study is the first study of rapid genomic testing primarily outside an intensive care setting and in a disease-specific cohort. We demonstrate feasibility of rapid genome sequencing in infants with new-onset epilepsy across multiple tertiary paediatric systems, with high diagnostic yield and clinical effect. Our findings provide support to prompt the use of state-of-the-art rapid genomic testing to facilitate early aetiological diagnosis that can inform urgent targeted management in this vulnerable population.

We demonstrate feasibility of expanding trio rapid genome sequencing from intensive care to outpatient and non-intensive care inpatient settings in four countries, with more than 80% of infants recruited from non-intensive care settings. More than 90% of parents consented, showing their interest in identifying the cause of their infant's seizures through early, rapid, and comprehensive genetic testing. Through the IPCHiP consortium, we harmonised study protocols across sites, strengthening the generalisability of our findings. Despite our sites having expertise in genomics and epilepsy, as well as institutional resources, this study posed challenges, including the cost of rapid genome sequencing and the need for sufficient personnel to efficiently achieve recruitment, research and clinical consent, sample collection, timely laboratory processes, variant interpretation, and return of results. Our experience highlights the need for collaboration between neurologists, geneticists, and genetic counsellors to ensure rapid identification of clinically significant variants to optimise patient care.

To our knowledge, this study is the first to evaluate rapid genome sequencing in infants with epilepsy. Our diagnostic yield of 43% is consistent with the yield of non-rapid genome sequencing (48%) in epilepsy reported in a recent systematic review (350 participants mostly with developmental and epileptic encephalopathies or severe phenotypes) and higher than that of chromosomal microarray (9%), gene panels (19%), and exome sequencing (24%), acknowledging that these studies have different inclusion or exclusion criteria.^4^ We excluded infants with acquired epilepsies, who would be predicted to have far lower likelihood of genetic aetiologies, and infants with known genetic causes, whose inclusion would have increased the diagnostic yield of rapid genome sequencing. Overall, although our cohort is not population based,^21^, ^22^ it represents most infants who present to tertiary paediatric centres with unexplained epilepsy. Most of our findings are de novo and could thus be relevant to patients of all ancestries. Nonetheless, a limitation of our study is that most infants have parent-reported White race. Future studies including more diverse populations are needed to achieve broader generalisability.

We confirm high diagnostic yield in neonatal-onset epilepsies, self-limited epilepsies, and early infantile developmental and epileptic encephalopathies, with relatively lower, although still important, yield in infantile epileptic spasms syndrome. The varied yield for different epilepsy syndromes highlights the importance of rigorous phenotyping when counselling families. Indeed, in four (29%) of 14 infants with primary findings of variants classified as uncertain significance by standardised criteria, the variants of uncertain significance were considered clinically diagnostic by expert clinicians given phenotype-genotype correlation; in two (50%) of four cases, further clinical investigation confirmed pathogenicity.

We also confirm genetic heterogeneity and the importance of channelopathies (15% of cohort) in infantile-onset epilepsies. In contrast to previous studies utilising gene panels or exome sequencing, we did not see clear predominance of a small number of genes (eg, *KCNQ2*, *PRRT2*, and *SCN1A*).^4^, ^7^, ^22^ This finding might reflect that previous studies using gene panels were limited to analysing specific subsets of genes or were conducted before the associations of other genes with epilepsy were identified. A potential limitation of our study is that our findings in a cohort of 100 participants might not reflect the full heterogenous genetic landscape of infantile-onset epilepsies. Furthermore, our study was not powered for a multivariate predictive model to assess which factors best predict a higher likelihood of identifying a genetic diagnosis; a larger cohort would be needed to develop such a model and investigate potential confounders in our analysis.

Genome sequencing represents the most comprehensive genetic testing approach but is not yet widely available. In most clinical settings, current standard of care includes chromosomal microarray or gene panel or exome sequencing (including tests performed on an exome sequencing or genome sequencing backbone using next-generation sequencing technology but analysed for only a small number of genes), performed concurrently or sequentially. Although our study was not designed to directly compare rapid genome sequencing with other tests, we demonstrate high yield of genome sequencing, performed as trio rapid genome sequencing whenever biological parents were available, and highlight its ability to detect genetic diagnoses not revealed by other modalities. Our findings support a genome-wide approach (exome sequencing or genome sequencing) as first-line genetic testing in infantile epilepsies, following guidelines endorsed by the American Epilepsy Society.^6^ Future studies are needed to accurately quantify the additional yield of genome sequencing compared with other tests in epilepsy.^23^ We anticipate that genome sequencing, which can detect single nucleotide variants, copy number variants, and other variant types, will become first-line testing and obviate the need for multiple tests in most patients, with trio rapid genome sequencing further enhancing yield.^24^

Infants with epilepsy represent a vulnerable population with substantial morbidity and mortality burden. Unlike previous epilepsy cohort studies with exome sequencing or genome sequencing performed in research laboratories,^25^, ^26^, ^27^ we performed rapid genome sequencing in clinically accredited laboratories, allowing immediate return of results to families and clinicians. Clinical utility was present for 55% of the cohort, including 98% with diagnostic rapid genome sequencing and 23% with non-diagnostic rapid genome sequencing or secondary or incidental findings. For participants with diagnostic rapid genome sequencing, we report a higher rate of clinical utility than with previous studies.^11^, ^12^, ^13^, ^14^, ^15^ Because of the current follow-up duration, we can only report short-term utility; additional utility is likely to be observed long term. We encourage future studies to report utility of non-diagnostic and secondary or incidental findings, as we found meaningful utility in multiple cases.

We identified numerous positive effects of early genetic diagnosis, affecting treatment (56%), evaluation (65%), and prognostic counselling (86%), and suggesting potential precision therapies (49%). In some cases, genetic diagnosis suggested a relatively good prognosis, with high likelihood of weaning antiseizure medication and normal development (eg, *PRRT2*). In other cases, genetic diagnosis suggested a relatively poor prognosis, with high likelihood of drug-resistant seizures, global developmental delay or intellectual disability, and even early mortality (eg, *BRAT1*), thus informing goals of care. Making a precise diagnosis also guides recurrence risk counselling, whether with inherited variants (high risk) or with apparently de novo variants (low but not zero risk due to the inability to detect parental gonadal mosaicism^28^), which is important to guide reproductive decision making for families.

We acknowledge some negative or difficult aspects of rapid genome sequencing. Early genetic diagnosis and awareness of future prognosis might contribute to diagnostic shock and parental stress.^29^, ^30^ As rapid genome sequencing becomes more widespread, families should be counselled before consenting and supported after results are returned. An important issue is the variable severity of conditions associated with a single gene. For example, *KCNQ2*, *KCNQ3*, *SCN1A*, *SCN2A*, and *SCN8A* are associated with phenotypes ranging from self-limited epilepsies with normal developmental outcome to intermediate severity conditions to drug-resistant epilepsies with profound developmental impairment.^2^ Precise prognostication is not always possible early on, and this uncertainty is very challenging for families. Our findings also included several neurodevelopmental disorders in which developmental impairments and other clinical features might become evident after infancy. Longitudinal evaluation is essential to monitor for additional clinical features and delineate the genetic landscape of epilepsy with and without neurodevelopmental disorders.^26^, ^31^

An additional area of uncertainty relates to the detection of variants of uncertain significance not considered clinically diagnostic, as was the case for 10% of our cohort, which might require time or additional investigation to resolve. Detection of variants of uncertain significance is a feature of all genomic tests given that our knowledge of the genome and disease associations is incomplete. Exome sequencing or genome sequencing, especially trio, might be associated with fewer variants of uncertain significance than gene panel testing.^32^

Finally, trio rapid genome sequencing can identify secondary or incidental diagnostic findings in the infant and, thus, parents, as occurred in 5% of our cohort. Parents require adequate pre-test counselling regarding this possibility and post-test support for coping with unexpected familial health implications.

The turnaround time of rapid genome sequencing has recently been reported to be on the order of hours in intensive care settings, compared with weeks in our study, suggesting room for improvement.^33^ However, given the inclusion of participants from both inpatient and outpatient settings, and the baseline lack of access to rapid—or any—genomic sequencing for many participants, a median turnaround time of 21 days from study enrolment to rapid genome sequencing result represents a major improvement over current standard of care. Moreover, although we aimed to perform trio genome sequencing for all individuals, for nine (9%) of 100 infants we were only able to perform duo or singleton genome sequencing. Although this approach might reduce opportunities for genetic diagnosis and discovery relative to trio testing, we believe these options are essential for ensuring equitable access when biological parents are unavailable.

We focused on initial diagnostic yield and short-term impact of rapid genome sequencing. Longitudinal follow-up will be essential to demonstrating the importance of rapid diagnosis in improving clinical, quality of life, and economic outcomes, which will inform advocacy and policy decisions about funding of genetic testing. Further aspects to assess include the parental perspective regarding rapid genome sequencing to ensure acceptability for those most likely to benefit from early diagnoses, reanalysis of genome sequencing data to increase diagnostic yield over time, and implementation of rapid genome sequencing into routine clinical practice.

We demonstrate the success and effect of a collaborative international model to provide rapid genetic diagnosis and clinical utility to infants with epilepsy through prospective enrolment, phenotyping, rapid genome sequencing, interpretation, and return of results. The diagnostic yield and short-term clinical effects are already high, and we anticipate long-term benefits for patients and families. As we shift the paradigm of epilepsy evaluation and diagnosis in the first year of life, this model might serve as a blueprint for advancing precision health for additional diseases whose aetiologies are suspected to be genetic but remain largely unexplained.

**This online publication has been corrected. The corrected version first appeared at thelancet.com/neurology on October 17, 2023**

## Data sharing

Deidentified clinical information included in this analysis and rapid genome sequencing results reported for each participant are provided in the supplementary material. Reported variants were deposited into public databases (eg, ClinVar) per the policies of the clinically accredited laboratories who performed the rapid genome sequencing. The study protocol is available after publication by request. Requests should be addressed to Amy McTague (a.mctague@ucl.ac.uk).

## Declaration of interests

APa is a current member of the National Institute of Health and Clinical excellence (NICE) technology appraisal committee B and has a financial interest in Genedrive PLC. KW has consulted for Stoke Therapeutics. JHC has received renumeration for lectures by GW pharma/Jazz, UCB/Zogenix, Biocodex, and Biogen; is a member of the Data Monitoring and Safety Committee for Admiral Trial (Stroke Therapeutics); and is Chair of the Medical Advisory Board for Matthews Friends, Dravet UK, and Hope for Hypothalamic Hamartoma. IES has served on scientific advisory boards for BioMarin, Chiesi, Eisai, Encoded Therapeutics, GlaxoSmithKline, Knopp Biosciences, Nutricia, Rogcon, Takeda Pharmaceuticals, UCB, and Xenon Pharmaceuticals; has received speaker honoraria from GlaxoSmithKline, UCB, BioMarin, Biocodex, Chiesi, Liva Nova, Nutricia, Zuellig Pharma, and Eisai; has received funding for travel from UCB, Biocodex, GlaxoSmithKline, Biomarin, Encoded Therapeutics, and Eisai; has served as an investigator for Anavex Life Sciences, Cerecin, Cerevel Therapeutics, Eisai, Encoded Therapeutics, EpiMinder, Epygenyx, ES-Therapeutics, GW Pharma, Marinus, Neurocrine BioSciences, Ovid Therapeutics, Takeda Pharmaceuticals, UCB, Ultragenyx, Xenon Pharmaceuticals, Zogenix, and Zynerba; and has consulted for Care Beyond Diagnosis, Epilepsy Consortium, Atheneum Partners, Ovid Therapeutics, UCB, Zynerba Pharmaceuticals, BioMarin, Encoded Therapeutics, and Biohaven Pharmaceuticals; and is a Non-Executive Director of Bellberry and a Director of the Australian Academy of Health and Medical Sciences and the Australian Council of Learned Academies. IES might accrue future revenue on pending patent WO61/010176 (filed in 2008; therapeutic compound); has a patent for *SCN1A* testing held by Bionomics and licensed to various diagnostic companies; has a patent molecular diagnostic or theranostic target for benign familial infantile epilepsy (PRRT2; 2011904493, 2012900190, and PCT/AU2012/001321 [TECH ID:2012-009]). GC has received honorarium from CADTH and serves as the Co-Lead of the Can-GARD Initiative and on the SickKids Precision Child Health steering committee. APo serves on the scientific advisory boards for TevardBio and Syngap Research Fund, and on the American Epilepsy Society Board of Directors. KBH has received support from RogCon Biosciences and Praxis Precision Medicines. AM has received consulting fees from Rocket Pharmaceuticals; honorarium from Jazz Pharmaceuticals; support for attending conferences from Jazz Pharmaceuticals and European Paediatric Neurology Society; fees for participating on boards for Biogen and Biocodex; and serves unpaid roles on the ILAE Genetic Literacy Task Force, EPICARE, and Great Ormond Street Hospital National Institute for Health and Care Research Biomedical Research Centre Chair of Junior Faculty. All other authors declare no competing interests.
