# Correction to *Lancet Neurol* 2023; 22: 812–25

**DOI:** 10.1016/S1474-4422(23)00411-8

**Published:** 2023-10-17

*D'Gama AM, Mulhern S, Sheidley BR, et al. Evaluation of the feasibility, diagnostic yield, and clinical utility of rapid genome sequencing in infantile epilepsy (Gene-STEPS): an international, multicentre, pilot cohort study.* Lancet Neurol *2023;* 22: *812–25*—In this Article, wording in some sections of figure 2A and in the key of figure 2D has been updated. Additionally, in the Findings section of the Summary, the fourth sentence should have read “…and 12 [30%] of 40 outpatient…”. These corrections have been made to the online version as of Oct 17, 2023.

