# Supplementary material for: Evaluation of the feasibility, diagnostic yield, and clinical utility of rapid genome sequencing in infantile epilepsy (Gene-STEPS): an international, multicentre, pilot cohort study
Source: Lancet Neurol. 2023 Sep;22(9):812–25. doi: 10.1016/S1474-4422(23)00246-6 (PMC11860300; doi:10.1016/S1474-4422(23)00246-6)
Supplement: Supplementary appendix [file mmc1.pdf]

THE LANCET
Neurology

Supplementary appendix

This appendix formed part of the original submission and has been peer reviewed. We post it as supplied by the authors.

Supplement to: D'Gama AM, Mulhern S, Sheidley BR, et al. Evaluation of the feasibility, diagnostic yield, and clinical utility of rapid genome sequencing in infantile epilepsy (Gene-STEPS): an international, multicentre, pilot cohort study. *Lancet Neurol* 2023; **22:** 812–25.

Supplementary Material Table of Contents

Supplementary Methods	2
Supplementary References	3
Supplementary Figure 1	4
Supplementary Table 1	5
Supplementary Table 2	6
Supplementary Table 3	9
Supplementary Table 4	14
Supplementary Table 5	18
Supplementary Table 6	25
Supplementary Table 7	27

Supplementary Methods: Rapid Genome Sequencing (GS) and Analysis

MCC (Victorian Clinical Genetics Services): GS was performed using Illumina Nextra DNA Flex or Illumina DNA PCR-Free library preparation followed by 2x151bp paired end sequencing on the Illumina NovaSeq6000 platform to an average depth of coverage of 30X. Genome alignment (build GRCh38/hg38 and Cambridge Reference Sequence mitochondrial genome) and variant calling was performed with Agilent Alissa Interpret analysis platform for SNVs and an in-house platform, CXGo, for CNVs. SNVs were analyzed using PanelApp Australia panels Malformations of cortical development_Superpanel, Regression, Intellectual disability syndromic and non-syndromic, Genetic Epilepsy, Metabolic Disorders Superpanel, Mendeliome, and Full Genome excluding Incidentalome (https://panelapp.agha.umccr.org/). Following phenotype driven analysis, a genome wide agnostic approach was taken to identify high impact/de novo/previously reported variants. Variants were classified using ACMG criteria[1]. Secondary findings identified in the proband were reported, and segregation information for these findings in parents were reported when available. Through the application of the virtual panels, the likelihood of incidental findings was reduced; however, incidental findings could be reported if identified at the discretion of the laboratory.

SickKids: GS was performed using Illumina TruSeq PCR-free library preparation followed by 2x150bp paired end sequencing on the Illumina Novaseq 6000 platform to an average depth of coverage of at least 35X. Genome alignment (build GRCh37/hg19) and small sequence variant (substitutions, insertions, duplications, and deletions) and copy number variant analysis was performed with the Illumina Dragen Germline analysis platform. Variants were classified using ACMG criteria[1]. Analysis of secondary findings was performed for the proband and variants in the genes recommended by the ACMG SF v3.0 or the medically actionable in childhood subset defined by Genome-Wide Sequencing Ontario (https://gsontario.ca/for-providers/secondary-findings/) were reported when they were known or expected to be pathogenic[2]. Variants in other disease genes that met the actionability criteria outlined in the ACMG policy statement may have been reported at the discretion of the Laboratory when identified (i.e., incidental findings). Information about segregation of secondary and incidental findings in relatives submitted for familial analysis was reported unless the family had opted out of receiving this information.

GOS ICH: GS was performed using Illumina PCR-free library preparation followed by 2x150 paired end sequencing on the Illumina Novaseq 6000 platform to an average depth of coverage of at least 35X. Genome alignment (build GRCh38/hg38) and variant calling was performed in parallel with the Illumina TruSight Software Suite and an in-house pipeline, which used the Illumina Dragen analysis platform, to identify SNVs and CNVs. SNVs were analyzed in parallel using a genetic epilepsy syndromes panel gene list (PanelApp v2.2: https://nhsgms-panelapp.genomicsengland.co.uk/panels/402/v2.2) and a genome-wide agnostic approach. Variants were classified using ACMG guidelines[1] and ACGS guidelines (https://www.acgs.uk.com/media/11631/uk-practice-guidelines-for-variant-classification-v4-01-2020.pdf). Unexpected findings (i.e., not related to epilepsy) in the proband were discussed in a multidisciplinary team and reported if deemed clinically actionable. Secondary findings in parents were not analyzed or reported unless they were identified during investigation of secondary findings in the proband.

BCH: GS and analysis was performed at GeneDx, Inc[3]. GS was performed using Illumina DNA PCR-Free Prep, Tagmentation library preparation followed by 2x150 paired end sequencing on the Illumina NovaSeq 6000 platform to an average depth of coverage of at least 40X. Genome alignment (build GRCh37/hg19) and variant calling was performed with the Illumina Dragen analysis platform and a custom-developed analysis tool (XomeAnalyzer) to identify sequence variants, repeat expansions in *FMR1* and *DMPK*, homozygous loss of *SMN1* exon 8, and most deletions and duplications greater than 1 kb in size. Variants were classified using ACMG criteria[1]. Reported clinically significant variants were confirmed by an appropriate orthogonal method in the proband and, if submitted, in selected relatives as necessary. If the family opted in to ACMG secondary findings, pathogenic and/or likely pathogenic variants in the genes recommended by the ACMG SF v3.0 were reported for the proband[2]. Reporting the presence or absence of the proband's identified secondary findings was available for relatives who underwent analysis as part of the proband's test. ACMG secondary findings were not reported for relatives who opted out of receiving ACMG secondary findings.

Supplementary References

1. Richards S, Aziz N, Bale S, et al. Standards and guidelines for the interpretation of sequence variants: a joint consensus recommendation of the American College of Medical Genetics and Genomics and the Association for Molecular Pathology. *Genet Med* 2015; **17**(5): 405-24.
2. Miller DT, Lee K, Chung WK, et al. ACMG SF v3.0 list for reporting of secondary findings in clinical exome and genome sequencing: a policy statement of the American College of Medical Genetics and Genomics (ACMG). *Genet Med* 2021; **23**(8): 1381-90.
3. Retterer K, Juusola J, Cho MT, et al. Clinical application of whole-exome sequencing across clinical indications. *Genet Med* 2016; **18**(7): 696-704.

Supplementary Figure 1: Study Time Intervals

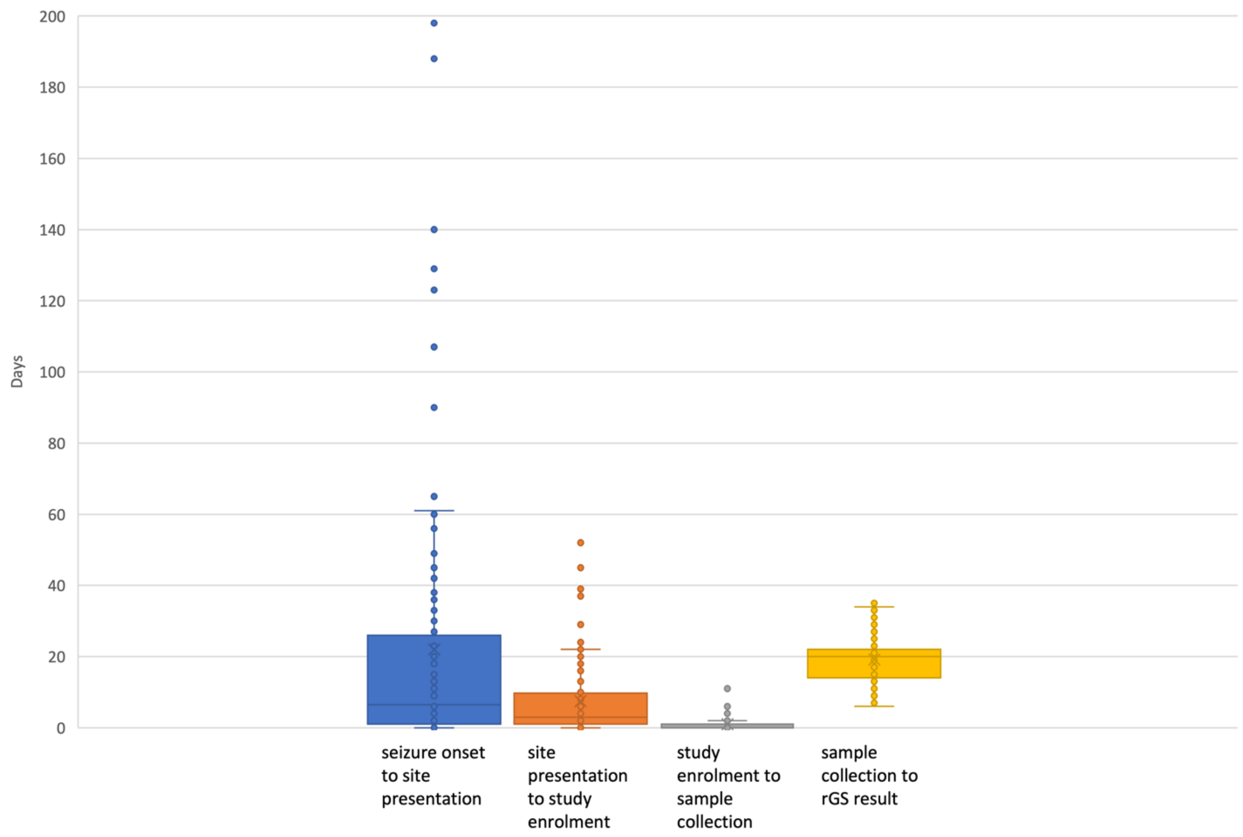

The time from seizure onset to rGS result can be broken down into four time intervals: seizure onset to site presentation, site presentation to study enrolment, study enrolment to sample collection, and sample collection to rGS results. The box-and-whisker plots display these four time intervals for the 100 study participants, highlighting the outliers in the first two time intervals (seizure onset to site presentation and site presentation to study enrolment).

Supplementary Table 1: Aggregate Demographics

	**N = 100**
**Centre**	
United States	34
Australia	22
Canada	22
United Kingdom	22
**Sex**	
Male	59
Female	41
**Parent-Reported Race**	
White	63
Asian	18
Multiple	8
Black	6
Middle Eastern	3
Other	2
**Referral source**	
Inpatient Unit (Non-Intensive Care)	43
Outpatient Unit	40
Neonatal Intensive Care Unit	13
Paediatric Intensive Care Unit	4

Supplementary Table 2: Demographics

ID	Sex	Gestational Age	Referral Source	Deceased in first year of life
001	Male	39w4d	NICU	No
002	Male	39w5d	Non-Intensive Care Inpatient	No
003	Female	40w0d	Non-Intensive Care Inpatient	No
004	Male	39w0d	Non-Intensive Care Inpatient	No
005	Female	41w2d	Non-Intensive Care Inpatient	No
006	Male	37w0d	Outpatient	No
007	Male	38w5d	Non-Intensive Care Inpatient	No
008	Female	37w0d	Outpatient	No
009	Male	38w0d	Non-Intensive Care Inpatient	No
010	Female	39w3d	Non-Intensive Care Inpatient	No
011	Male	38w4d	Non-Intensive Care Inpatient	No
012	Female	39w6d	PICU	No
013	Male	36w6d	NICU	Yes
014	Female	39w2d	Non-Intensive Care Inpatient	No
015	Male	Term	Non-Intensive Care Inpatient	No
016	Male	33w1d	NICU	Yes
017	Female	40w3d	Outpatient	No
018	Female	39w1d	Non-Intensive Care Inpatient	Yes
019	Male	38w5d	Non-Intensive Care Inpatient	No
020	Male	39w0d	Outpatient	No
021	Female	37w0d	Outpatient	No
022	Male	38w5d	Outpatient	No
023	Male	39w0d	Non-Intensive Care Inpatient	No
024	Male	38w5d	Non-Intensive Care Inpatient	No
025	Female	41w1d	NICU	No
026	Male	39w5d	Non-Intensive Care Inpatient	No
027	Female	39w2d	NICU	No
028	Female	33w4d	NICU	No
029	Male	39w0d	NICU	Yes
030	Male	36w3d	PICU	No
031	Male	Term	Non-Intensive Care Inpatient	No
032	Female	37w6d	Non-Intensive Care Inpatient	No
033	Male	29w1d	Non-Intensive Care Inpatient	No
034	Female	38w0d	NICU	No
035	Male	40w	Outpatient	No
036	Male	35w5d	Outpatient	No
037	Male	31w6d	NICU	Yes
038	Female	39w1d	Outpatient	No
039	Male	42w	Outpatient	No
040	Male	40w	Outpatient	No
041	Male	39w	Non-Intensive Care Inpatient	No
042	Female	39w	Outpatient	No
043	Female	40w	Outpatient	No
044	Male	40w	Non-Intensive Care Inpatient	No

045	Male	39w	Outpatient	No
046	Female	40w6d	Outpatient	No
047	Male	40w	Outpatient	No
048	Female	40w	Outpatient	No
049	Male	40w	Outpatient	No
050	Female	40w	Non-Intensive Care Inpatient	No
051	Male	40w	Non-Intensive Care Inpatient	No
052	Female	42w	Outpatient	No
053	Male	38w6d	Outpatient	No
054	Male	36w2d	Outpatient	No
055	Female	40w	Outpatient	No
056	Female	40w	Outpatient	No
057	Female	35w3d	Outpatient	No
058	Male	38w	Non-Intensive Care Inpatient	No
059	Male	37w3d	Non-Intensive Care Inpatient	No
060	Male	38w	Non-Intensive Care Inpatient	No
061	Female	38w3d	Non-Intensive Care Inpatient	No
062	Male	34w	Non-Intensive Care Inpatient	No
063	Male	Term	Outpatient	No
064	Female	35w	Non-Intensive Care Inpatient	No
065	Female	38w3d	Non-Intensive Care Inpatient	No
066	Female	41w	Outpatient	No
067	Female	38w5d	Outpatient	No
068	Female	26w	Non-Intensive Care Inpatient	No
069	Male	38w3d	Non-Intensive Care Inpatient	No
070	Male	41w	Non-Intensive Care Inpatient	No
071	Female	40w	Non-Intensive Care Inpatient	No
072	Male	40w2d	Outpatient	No
073	Male	38w	Non-Intensive Care Inpatient	No
074	Male	40w	PICU	No
075	Male	41w3d	PICU	No
076	Male	40w	Non-Intensive Care Inpatient	No
077	Male	37w5d	Outpatient	No
078	Male	39w	Non-Intensive Care Inpatient	No
079	Male	38w3d	Non-Intensive Care Inpatient	No
080	Male	38w6d	Non-Intensive Care Inpatient	No
081	Male	38w1d	Outpatient	No
082	Female	40w3d	Outpatient	No
083	Male	41w1d	Outpatient	No
084	Male	37w2d	Non-Intensive Care Inpatient	No
085	Male	40w3d	NICU	Yes
086	Female	39w0d	Non-Intensive Care Inpatient	No
087	Female	36w3d	Non-Intensive Care Inpatient	No
088	Female	37w6d	Outpatient	No
089	Female	39w5d	Outpatient	No
090	Female	34w0d	Outpatient	No
091	Female	37w5d	Outpatient	No

092	Male	40w3d	NICU	No
093	Male	37w0d	Non-Intensive Care Inpatient	No
094	Male	39w0d	Outpatient	No
095	Male	40w4d	Outpatient	No
096	Female	39w3d	Non-Intensive Care Inpatient	No
097	Female	39w5d	Outpatient	No
098	Male	Term	Outpatient	No
099	Male	32w3d	NICU	No
100	Female	39w2d	NICU	No

N/PICU: Neonatal/Paediatric Intensive Care Unit

Supplementary Table 3: Clinical Presentation

ID	Age at seizure onset (days)	Seizure type at onset	Epilepsy syndrome at onset	Developmental delay prior to onset (N/A for neonates up to 44w PMA)	Developmental plateau/regression following onset (N/A for neonates up to 44w PMA)	Malformation of Cortical Development on MRI	Other clinical features	Family history of seizures (first or second degree relative)	Parental consanguinity
001	4	Focal	EIDEE	N/A	N/A	Left sided polymicrogyria	-	Maternal grandfather with epilepsy	No
002	224	Generalized	IESS	Yes	No	No	Hypotonia, infantile sleep myoclonus	No	No
003	361	Focal	Focal Epilepsy	No	No	No	-	No	No
004	40	Focal	Focal Epilepsy	No	No	No	-	No	No
005	46	Focal	EIDEE	No	No	No	-	No	No
006	188	Focal	Focal Epilepsy	No	No	No	-	Paternal grandmother with epilepsy	No
007	11	Focal	Focal Epilepsy	N/A	N/A	No	-	No	No
008	327	Generalized	IESS	No	No	No	-	Paternal uncle with epilepsy (adult onset)	No
009	119	Focal	Focal Epilepsy	No	No	No	-	Mother with seizures in adolescence; maternal half-sister with epilepsy, focal cortical dysplasia, and de novo *DEPDC5* pathogenic variant; maternal grandmother with seizures	No
010	145	Focal	Focal Epilepsy	No	No	No	Macrocephaly	No	No
011	42	Both	EIDEE	Yes	No	Microcephaly with simplified gyral pattern with anterior to posterior gradient	Microcephaly, strabismus	No	No
012	1	Focal	Focal Epilepsy	N/A	N/A	No	Hypotonia	No	No
013	0	Focal	EIDEE	N/A	N/A	Aqueductal stenosis, ventriculomegaly, polymicrogyria, gray matter band heterotopia, dysmorphic cerebellum with vermian hypoplasia, foreshortened and thinned corpus callosum, absence of the olfactory bulbs	Hypotonia, IUGR, dysmorphic features, congenital heart disease	No	No
014	64	Both	EIDEE	No	No	No	-	No	No
015	149	Generalized	IESS	No	No	Polymicrogyria	Macrocephaly	No	No

016	1	Focal	Focal Epilepsy	N/A	N/A	No	Hypotonia, arthrogryposis multiplex congenita	No	No
017	192	Focal	IESS	Yes	No	No	-	Paternal half uncle with epilepsy	No
018	123	Focal	"Severe" DS	Yes	No	No	Microcephaly, hypotonia, cardiac anatomical variant, bilateral dislocated hips with dysplastic acetabulum	Brother with febrile seizure as a toddler	No
019	162	Both	IESS	No	Yes	No	Hypotonia, IUGR, congenital heart disease, feeding difficulties	No	Yes
020	78	Generalized	IESS	No	No	No	-	Paternal uncle with febrile seizures as an infant	No
021	291	Focal	SeLIE	No	No	No	-	No	No
022	114	Generalized	IESS	No	No	No	-	No	No
023	183	Generalized	IESS	Yes	No	No	-	No	No
024	164	Focal	Focal Epilepsy	No	No	Left occipitotemporal malformation (Focal Cortical Dysplasia)	Congenital hypothyroidism	Maternal aunt with seizure in adolescence	No
025	0	Focal	EIDEE	N/A	N/A	No	Dysmorphic features	No	Yes
026	251	Generalized	IESS	Yes	Yes	No	Hypotonia, dysmorphic features	No	No
027	1	Focal	Focal Epilepsy	N/A	N/A	No	Hypertonia, dysmorphic features, feeding difficulties	No	No
028	35	Focal	SeLNE	N/A	N/A	No	Feeding difficulties	No	No
029	10	Focal	EIDEE	N/A	N/A	Callosal dysgenesis with hypoplasia of the splenium	Hypotonia, dysmorphic features, Pierre Robin sequence, left renal collecting system dilation, feeding difficulties	No	No
030	48	Focal	EIDEE	N/A	N/A	No	Dysmorphic features, feeding difficulties	No	Yes
031	82	Focal	Focal Epilepsy	No	No	No	Dysmorphic features	Maternal half uncle with febrile seizures as a child	No
032	126	Focal	Focal Epilepsy	No	No	No	-	No	No
033	238	Generalized	IESS	No	No	No	SVT, feeding difficulties	No	No
034	115	Focal	SeLIE	No	No	No	-	Father with seizures from birth until 9 months of age	No
035	66	Focal	Focal Epilepsy	No	No	No	-	No	No
036	90	Both	EIDEE	Yes	Yes	No	Hypotonia	No	No
037	18	Focal	EIDEE	N/A	N/A	No	Hypotonia, dysmorphic features, autonomic instability	No	No
038	151	Generalized	IESS	No	No	No	-	No	No

039	121	Generalized	Other	No	Yes	No	Cow's milk protein intolerance	Mother with febrile seizures	No
040	227	Focal	Other	No	No	No	-	Maternal aunt with seizures, maternal grandmother with seizure	No
041	99	Both	SeLIE	No	No	No	-	No	No
042	192	Generalized	IESS	No	Yes	No	-	Paternal grandfather with epilepsy (adolescent onset)	No
043	202	Generalized	IESS	No	Yes	No	-	No	No
044	58	Generalized	Other	No	No	No	Posterior urethral valves and hydroureteronephrosis	Father with epilepsy (infantile onset)	No
045	206	Generalized	Other	No	Yes	No	Axial hypotonia, lower extremity hypertonia	No	No
046	16	Focal	Other	N/A	N/A	No	-	No	No
047	196	Generalized	IESS	Yes	Yes	No	-	No	No
048	162	Generalized	IESS	No	No	No	-	Both parents and sister with febrile seizures	No
049	153	Generalized	IESS	No	Yes	No	Strabismus, laryngomalacia	No	No
050	138	Generalized	Other DEE	Yes	Yes	No	Dystonia, bilateral optic atrophy	Paternal grandfather and uncle with seizures	Yes
051	129	Focal	IESS	No	Yes	Bilateral frontal polymicrogyria with callosal dysplasia	Vitamin B12 deficiency	Paternal uncle with epilepsy (adult onset)	No
052	27	Generalized	Other DEE	No	Yes	No	-	No	No
053	236	Generalized	IESS	No	Yes	No	-	No	No
054	86	Focal	IESS	Yes	Yes	No	Dyskinesia	No	No
055	145	Focal	Other DEE	No	Yes	No	-	No	No
056	108	Focal	Other DEE	No	Yes	No	-	No	No
057	161	Unknown	IESS	No	Yes	No	-	No	No
058	53	Focal	Focal Epilepsy	No	No	Multiple bottom-of-sulcus dysplasia-like dysplasias	-	No	No
059	144	Focal	Focal Epilepsy	No	No	No	-	No	No
060	146	Focal	SeLIE	No	No	No	-	Mother with suspected febrile seizure as an infant	No
061	114	Focal	Focal Epilepsy	Yes	No	No	Developmental dysplasia of the hips	No	No
062	169	Unknown	IESS	No	Yes	No	Symmetric IUGR, inguinal hernia, neonatal cholestasis	No	No
063	243	Focal	Focal Epilepsy	Yes	No	No	Vitamin B12 deficiency	No	No
064	154	Unknown	Complex febrile seizures	No	No	No	-	No	No
065	0	Focal	SeLNE	N/A	N/A	No	Congenital heart disease	No	No

066	200	Focal	Focal Epilepsy	Yes	No	No	Hypotonia	No	No
067	326	Focal	Complex febrile seizures	Yes	No	No	Hypotonia, macrocephaly	No	No
068	278	Unknown	IESS	Yes	Yes	No	Hypotonia, complications of prematurity	No	No
069	42	Focal	Focal Epilepsy	No	No	No	-	No	No
070	68	Focal	Focal Epilepsy	No	No	No	-	Maternal grandmother with epilepsy (adult onset)	No
071	29	Focal	Focal Epilepsy	No	No	No	-	No	No
072	251	Unknown	IESS	No	Yes	No	-	No	No
073	209	Focal	SeLIE	No	No	No	-	No	No
074	251	Both	DS	No	No	No	-	Mother and maternal aunt with febrile seizures as children	No
075	262	Focal	Complex febrile seizures	No	No	No	-	Maternal uncle with febrile seizures as a child	No
076	1	Focal	Focal Epilepsy	N/A	N/A	Cerebral dysgyria, absent septum pellucidum, dysmorphic and enlarged basal ganglia and lateral ventricles	Axial hypotonia, appendicular hypertonia, hyperkinetic movement disorder, microcephaly	No	No
077	144	Focal	Focal Epilepsy	No	No	Unclear	-	Paternal grandmother with suspected seizure	No
078	163	Generalized	IESS	Yes	Yes	No	Hypotonia, hemangiomas	No	No
079	46	Focal	Focal Epilepsy	No	No	No	Nystagmus	No	Yes
080	221	Both	Other	No	No	Left hemimegalencephaly, dysmorphic corpus callosum	-	No	No
081	180	Generalized	IESS	No	No	No	-	No	No
082	2	Generalized	SeLNE	N/A	N/A	No	-	Brother with febrile seizures	No
083	134	Generalized	IESS	No	No	No	-	No	No
084	119	Generalized	SeLIE	No	No	No	-	Maternal uncle with seizures	No
085	4	Generalized	EIDEE	N/A	N/A	No	Hypotonia, apnea	No	No
086	243	Focal	SeLIE	Yes	No	No	Hypotonia	No	No
087	39	Generalized	SeLIE	No	No	No	-	Paternal grandmother with seizures	No
088	105	Generalized	IESS	Yes	Yes	No	-	No	No

089	175	Generalized	IESS	No	No	No	-	No	No
090	204	Generalized	IESS	Yes	No	No	Hypotonia	No	No
091	152	Focal	SeLIE	No	No	No	Macrocephaly	Father with febrile seizures	No
092	5	Focal	EIDEE	N/A	N/A	No	Hypotonia, macrocephaly, bilateral talipes equinovarus, congenital heart disease	No	No
093	189	Generalized	SeLIE	No	No	No	-	Mother with seizures	No
094	234	Generalized	IESS	Yes	Yes	Septo-optic dysplasia, right frontal polymicrogyria, Chiari malformation	Hypotonia, septo-optic dysplasia, obesity	No	No
095	47	Generalized	EIDEE	No	Yes	No	Head lag	No	No
096	82	Unknown	SeLIE	No	No	No	-	Maternal grandmother with epilepsy	No
097	127	Generalized	IESS	No	No	No	Hypotonia	No	No
098	203	Generalized	IESS	No	Yes	No	Hypotonia	No	No
099	10	Unknown	Other	N/A	N/A	No	Abnormal tone, central apnea	No	Yes
100	4	Unknown	SeLFNIE	N/A	N/A	No	-	Father and paternal uncle with neonatal seizures	No

DS: Dravet Syndrome, EIDEE: Early Infantile Developmental and Epileptic Encephalopathy, IESS: Infantile Epileptic Spasms Syndrome, IUGR: intrauterine growth restriction, MRI: magnetic resonance imaging, N/A: not applicable, PMA: postmenstrual age, SeLFNIE: Self-Limited Familial Neonatal-Infantile Epilepsy, SeLIE: Self-Limited Infantile Epilepsy, SeLNE: Self-Limited Neonatal Epilepsy, SVT: supraventricular tachycardia

Supplementary Table 4: Previous or Concurrent Genetic Testing and Timing to Genome Sequencing

ID	Other genetic testing (non-diagnostic unless otherwise noted)	Age at first presentation with seizures to study site (days)	Age at Gene-STEPS enrollment (days)	Age at proband sample collection (days)	Type of genome sequencing	Age at GS report (days)
001	Prior CMA	7	11	11	Trio	26
002	Concurrent CMA, panel	226	227	228	Trio	241
003	Concurrent CMA	376	378	378	Trio	385
004	No	50	66	67	Trio	77
005	No	51	53	53	Trio	72
006	Prior panel	189	226	230	Trio	241
007	No	11	15	18	Trio	29
008	No	330	343	345	Trio	359
009	No	119	156	157	Trio	171
010	No	145	162	162	Trio	179
011	No	107	108	109	Trio	120
012	Prior panel	1	5	5	Trio	22
013	Prior prenatal karyotype, CMA	0	4	5	Trio	25
014	Concurrent CMA	64	72	72	Trio	81
015	No	149	151	151	Trio	161
016	Prior CMA, ES, mito	1	25	27	Trio	37
017	Prior panel	207	221	221	Trio	237
018	No	123	125	126	Trio	155
019	Prior CMA	163	165	165	Trio	172
020	Prior panel	114	114	121	Trio	127
021	No	292	344	345	Trio	357
022	Prior CMA, panel	144	144	144	Trio	173
023	No	189	192	192	Trio	204
024	No	202	206	207	Trio	214
025	Prior rapid ES	0	4	5	Trio	40
026	No	253	256	257	Trio	271
027	No	1	5	5	Trio	20
028	No	35	43	44	Trio	58

029	Prior prenatal karyotype, CMA	13	15	15	Trio	28
030	Prior CMA	55	58	59	Trio	73
031	No	91	99	110	Trio	121
032	No	126	133	135	Trio	155
033	No	239	253	253	Trio	260
034	No	115	118	118	Trio	139
035	Concurrent CMA, panel (identified; trio GS r59 panel [https://panelapp.genomicsengland.co.uk/panels/489/])	123	123	123	Trio	144
036	Concurrent CMA, panel (trio GS r59 panel [https://panelapp.genomicsengland.co.uk/panels/489/])	106	106	106	Trio	126
037	Concurrent CMA, subsequent single gene *BRAT1* testing (for validation)	18	47	51	Trio	61
038	Concurrent CMA, panel (trio GS r59 panel [https://panelapp.genomicsengland.co.uk/panels/489/])	291	291	291	Trio	314
039	Concurrent CMA, panel (identified; trio GS r59 panel [https://panelapp.genomicsengland.co.uk/panels/489/])	319	364	364	Trio	386
040	Concurrent CMA, panel (trio GS r59 panel [https://panelapp.genomicsengland.co.uk/panels/489/])	241	243	243	Trio	265
041	Concurrent panel (identified; trio GS r59 panel [https://panelapp.genomicsengland.co.uk/panels/489/])	103	105	109	Trio	130
042	Concurrent CMA, panel (trio GS r59 panel [https://panelapp.genomicsengland.co.uk/panels/489/])	248	248	248	Trio	269
043	Concurrent CMA, panel (trio GS r59 panel [https://panelapp.genomicsengland.co.uk/panels/489/])	247	247	247	Trio	271
044	Concurrent panel (trio GS r59 panel [https://panelapp.genomicsengland.co.uk/panels/489/])	64	70	70	Trio	90
045	Concurrent panel (trio GS r59 panel [https://panelapp.genomicsengland.co.uk/panels/489/])	220	230	234	Trio	266
046	Concurrent CMA (identified as mosaic), panel (identified; trio GS r59 panel [https://panelapp.genomicsengland.co.uk/panels/489/])	20	20	20	Trio	47
047	Concurrent CMA, panel (trio GS r59 panel [https://panelapp.genomicsengland.co.uk/panels/489/])	199	199	199	Trio	219
048	Concurrent panel (trio GS r59 panel [https://panelapp.genomicsengland.co.uk/panels/489/])	180	180	180	Trio	211
049	Concurrent panel (trio GS r59 panel [https://panelapp.genomicsengland.co.uk/panels/489/])	180	180	180	Trio	209
050	Concurrent CMA, trio ES (identified)	140	142	149	Trio	160
051	Concurrent CMA, panel (trio GS r59 panel [https://panelapp.genomicsengland.co.uk/panels/489/])	136	149	149	Trio	158
052	Concurrent CMA, panel (identified; trio GS r59 panel [https://panelapp.genomicsengland.co.uk/panels/489/])	41	41	41	Trio	52
053	Concurrent CMA, panel (trio GS r59 panel [https://panelapp.genomicsengland.co.uk/panels/489/])	297	297	297	Trio	319
054	Concurrent CMA, panel (identified; trio GS r59 panel [https://panelapp.genomicsengland.co.uk/panels/489/])	119	119	119	Trio	128
055	Concurrent CMA, panel (trio GS r59 panel [https://panelapp.genomicsengland.co.uk/panels/489/])	159	159	159	Trio	180
056	Concurrent CMA, panel (identified; trio GS r59 panel [https://panelapp.genomicsengland.co.uk/panels/489/])	150	150	150	Trio	167
057	No	167	188	188	Trio	209
058	No	63	85	88	Trio	110
059	Concurrent CMA	154	156	156	Trio	178
060	No	147	149	149	Trio	169
061	Concurrent CMA (identified)	114	118	118	Trio	140

062	Concurrent CMA	215	217	217	Trio	228
063	Concurrent karyotype, CMA	271	310	310	Duo	324
064	No	342	343	344	Duo	360
065	No	2	24	24	Duo	53
066	CMA	213	213	213	Trio	233
067	Prior CMA, Fragile X testing	326	350	350	Trio	362
068	Concurrent CMA	281	294	294	Trio	315
069	No	91	111	111	Trio	133
070	Concurrent CMA	69	72	72	Trio	87
071	No	43	45	45	Trio	61
072	Concurrent CMA	282	296	296	Trio	318
073	Subsequent CMA (for validation)	209	213	213	Duo	235
074	No	251	269	269	Trio	287
075	No	262	263	264	Trio	285
076	No	61	64	64	Trio	99
077	No	162	162	162	Trio	183
078	Concurrent CMA, subsequent karyotype (for validation, and identified as mosaic)	197	200	200	Singleton	227
079	Concurrent CMA	153	161	161	Trio	186
080	Concurrent CMA	222	225	225	Duo	247
081	Concurrent CMA	195	196	208	Trio	243
082	Concurrent panel (identified)	62	62	63	Trio	91
083	Concurrent CMA	157	157	157	Trio	179
084	Concurrent CMA	120	124	124	Trio	145
085	Concurrent panel	4	9	15	Trio	42
086	Concurrent CMA, panel	259	261	265	Trio	287
087	No	46	48	49	Trio	76
088	Concurrent CMA	228	250	250	Trio	275
089	Concurrent CMA	196	196	197	Duo	230
090	Concurrent CMA	224	231	231	Trio	265
091	Concurrent CMA (identified), panel	242	242	242	Trio	274
092	Concurrent CMA, panel (identified but as VUS)	5	9	9	Trio	45
093	No	189	192	192	Trio	213
094	Concurrent CMA	245	258	258	Trio	273
095	Concurrent CMA	70	88	95	Duo	111

096	No	84	86	86	Duo	103
097	Concurrent CMA	132	134	134	Trio	159
098	Concurrent CMA	332	332	333	Trio	352
099	Concurrent panel	10	19	22	Trio	39
100	Concurrent panel (identified)	5	8	8	Trio	24

CMA: chromosomal microarray, ES: exome sequencing, GS: genome sequencing, mito: mitochondrial sequencing

Supplementary Table 5: Primary Genome Sequencing Results (for Epilepsy Phenotype)

ID	Primary GS Results (for epilepsy phenotype)	Gene symbol and/or cytoband	Variant (g. nomenclature; hg19)	Variant (c. nomenclature)	Variant (p. nomenclature)	Zygosity	Inheritance	Associated disease	Mode of Inheritance	Classification
001	Diagnostic	*DEPDC5*	chr22:g.32242859delA	NM_001242896.1:c.3061del	p.(Ile1021Phefs*58)	Heterozygous	Maternal	DEPDC5-Related Disorder	Autosomal Dominant	Pathogenic
002	Diagnostic	*DYNC1H1*	chr14:g.102478782G>A	NM_001376.4:c.6989G>A	p.(Gly2330Glu)	Heterozygous	De novo	DYNC1H1-Related Disorder	Autosomal Dominant	Likely Pathogenic
003	Non-diagnostic									
004	Non-diagnostic (Carrier)	*PNKP*	chr19:g.50365626A>G	NM_007254.4:c.1029+2T>C	p.(?)	Heterozygous	Paternal	PNPK-Related Disorder	Autosomal Recessive	Pathogenic
005	Non-diagnostic (VUS)	*RERE*	chr1:g.8419896_8419901dupTCGCTC	NM_012102.3:c.3546_3551dup	p.(Arg1184_Glu1185dup)	Heterozygous	De Novo	RERE-Related Disorder	Autosomal Dominant	VUS
006	Non-diagnostic									
007	Non-diagnostic									
008	Non-diagnostic (VUS)	*HCN2*	chr19:g.[613454G>C];[616161G>C]	NM_001194.3:c.[1791G>C];[2357G>C]	p.[(Lys597Asn)];[(Ser786Thr)]	Compound heterozygous	Paternal and Maternal	HCN2-Related Disorder	Autosomal Dominant	VUS
009	Non-diagnostic									
010	Non-diagnostic (VUS)	*PTEN*	chr10:g.89693004A>G	NM_000314.4:c.488A>G	p.(Lys163Arg)	Heterozygous	Maternal	PTEN-Related Hamartoma Tumor Syndrome	Autosomal Dominant	VUS
011	Diagnostic	*TNPO2*	chr19:g.12826319C>T	NM_001136196.1:c.466G>A	p.(Asp156Asn)	Heterozygous	De novo	TNPO2-Related Disorder	Autosomal Dominant	Pathogenic
012	Non-diagnostic (VUS)	*LRP2*	chr2:g.[170097585G>A];[170070235C>T]	NM_004525.2:c.[3958C>T];[5972G>A]	p.[(Leu1320Phe)];[(Gly1991Glu)]	Compound heterozygous	Paternal and Maternal	LRP2-Related Disorder	Autosomal Recessive	VUS
013	Non-diagnostic									
014	Diagnostic	*PPP3CA*	chr4:g.101953510_101953511delTC	NM_000944.4:c.1255_1256del	p.(Ser419Cysfs*31)	Heterozygous	De novo	PPP3CA-Related Disorder	Autosomal Dominant	Pathogenic
015	Diagnostic	*PTEN*	chr10:g.89624275C>T	NM_000314.4:c.49C>T	p.(Gln17*)	Heterozygous	De novo	PTEN-Related Hamartoma Tumor Syndrome	Autosomal Dominant	Pathogenic

016	Diagnostic	*DMPK*	-	NM_004409.5:c.*224_226CTG[>200]; confirmed >2000 on subsequent Southern blot	-	Heterozygous	Maternal	DMPK-Related Myotonic Dystrophy	Autosomal Dominant	Pathogenic
017	Non-diagnostic									
018	Diagnostic	2q24.2q24.3	chr2:g.161682102_167003129del	-	-	Heterozygous	De novo			Pathogenic
019	Non-diagnostic									
020	Non-diagnostic									
021	Diagnostic	*SCN8A*	chr12:g.52174539G>A	NM_014191.3:c.3926G>A	p.(Arg1309Gln)	Heterozygous	De Novo	SCN8A-Related Epilepsy	Autosomal Dominant	Pathogenic
022	Non-diagnostic									
023	Non-diagnostic (VUS)	*PLXNA3*	chrX:g.153696476A>G	NM_017514.3:c.3872A>G	p.(Glu1291Gly)	Hemizygous	Maternal	None Currently Described	Unknown	VUS
024	Non-diagnostic									
025	Non-diagnostic									
026	Diagnostic	*SETD5*	chr3:g.9489554T>G	NM_001080517.1:c.1967T>G	p.(Leu656*)	Heterozygous	De Novo	SETD5-Related Neurodevel-opmental Disorder with Multiple Anomalies	Autosomal Dominant	Pathogenic
027	Diagnostic	*ZC4H2*	chrX:g.64139038delG	NM_018684.3:c.450del	p.(Ile151Serfs*36)	Heterozygous	De Novo	ZC4H2-Related Neurodevel-opmental Disorder with Multiple Anomalies	X-Linked	Pathogenic
028	Diagnostic	*SCN2A*	chr2:g.166165731T>C	NM_001040143.1:c.662T>C	p.(Val221Ala)	Heterozygous	De Novo	SCN2A-Related Disorder	Autosomal Dominant	Likely Pathogenic
029	Diagnostic	*MOGS*	chr2:g.[74689455C>G];[74688446C>T]	NM_006302.2:c.[1461G>C];[2470G>A]	p.[(Glu487Asp)];[(Gly824Ser)]	Compound heterozygous	Maternal and De novo	Congenital Disorder of Glycosyla-tion Type IIb	Autosomal Recessive	Likely Pathogenic/ VUS (confirmed biochemically)

030	Diagnostic	*MOCS2*	chr5:g.52397927C>T	NM_004531.5:c.226G>A	p.(Gly76Arg)	Homozygous	Maternal and Paternal	MOCS2-Related Molybdenum Cofactor Deficiency	Autosomal Recessive	Pathogenic
031	Non-diagnostic (VUS)	*SCN3A*	chr2:g.165947112C>T	NM_006922.3:c.5551G>A	p.(Asp1851Asn)	Heterozygous	Maternal	SCN3A-Related Disorder	Autosomal Dominant	VUS
032	Diagnostic	*CSNK2B*	chr6:g.31637285G>A	NM_001320.6:c.557G>A	p.(Arg186Lys)	Heterozygous	De Novo	CSNK2B-Related Neurodevel-opmental Disorder	Autosomal Dominant	Likely Pathogenic
033	Non-diagnostic									
034	Diagnostic	*SCN2A*	chr2:g.166245603T>A	NM_021007.2:c.5287T>A	p.(Ser1763Thr)	Heterozygous	Paternal	SCN2A-Related Disorder	Autosomal Dominant	VUS (considered clinically diagnostic)
035	Diagnostic	*STXBP1*	chr9:g.130422322T>C	NM_003165.3:c.260T>C	p.(Leu87Pro)	Heterozygous	De novo	DEE 4	Autosomal Dominant	Likely Pathogenic
036	Non-diagnostic									
037	Diagnostic	*BRAT1*	chr7:g.[2579182C>T];[2584679dupT]	NM_152743.4:c.[1593G>A];[294dup]	p.[(Trp531*)];[(Leu99Thrfs*92)]	Compound heterozygous	Maternal and Paternal	Lethal Neonatal Rigidity and Multifocal Seizure Syndrome	Autosomal Recessive	Pathogenic
038	Non-diagnostic									
039	Diagnostic	*GABRB3*	chr15:g.27017551T>C	NM_000814.5:c.238A>G	p.(Met80Val)	Heterozygous	De novo	DEE 43	Autosomal Dominant	Likely Pathogenic
040	Non-diagnostic									
041	Diagnostic	*KCNQ2*	chr20:g.62070003C>T	NM_172107.2:c.998G>A	p.(Arg333Gln)	Heterozygous	De novo	Benign Neonatal Seizures 1	Autosomal Dominant	Pathogenic
042	Non-diagnostic									
043	Non-diagnostic									
044	Non-diagnostic									

045	Diagnostic	*POLR3B*	chr12:g.106827634G>A	NM_018082.5:c.1765G>A	p.(Gly589Arg)	Heterozygous	De novo	POLR3B-Related Disease	Autosomal Dominant/ Autosomal Recessive	VUS (considered clinically diagnostic; subsequently had abnormal nerve conduction testing)
046	Diagnostic	2q24.3q32.1	chr2:g.164436654_185107319dup	-	-	Heterozygous	De novo mosaic		Autosomal Dominant	Pathogenic
047	Non-diagnostic									
048	Non-diagnostic									
049	Non-diagnostic									
050	Diagnostic	*ATP6V1A*	chr3:g.113507604G>A	NM_001690.3:c.761G>A	p.(Cys254Tyr)	Heterozygous	De novo	DEE 93	Autosomal Dominant	Likely Pathogenic
051	Non-diagnostic									
052	Diagnostic	*CDKL5*	chrX:g.18622692C>T	NM_001323289.2:c.1648C>T	p.(Arg550*)	Heterozygous	De novo	DEE 2	X-linked	Pathogenic
053	Non-diagnostic									
054	Diagnostic	*STXBP1*	chr9:g.130428484C>T	NM_003165.4:c.703C>T	p.(Arg235*)	Heterozygous	De novo	DEE 4	Autosomal Dominant	Pathogenic
055	Non-diagnostic									
056	Diagnostic	*SCN8A*	chr12:g.52200885G>A	NM_014191.3:c.5615G>A	p.(Arg1872Gln)	Heterozygous	De novo	DEE 13	Autosomal Dominant	Pathogenic
057	Non-diagnostic									
058	Diagnostic	*TSC2*	chr16:g.2130366C>T	NM_000548.3:c.3598C>T	p.(Arg1200Trp)	Heterozygous	Maternal	Tuberous sclerosis 2	Autosomal Dominant	Pathogenic
059	Non-diagnostic (VUS)	*CACNA1A*	chr19:g.13443725C>T	NM_001127222.1:c.1213G>A	p.(Ala405Thr)	Heterozygous	Paternal	DEE 42	Autosomal Dominant	VUS
060	Diagnostic	*PRRT2*	chr16:g.29825024dupC	NM_145239.2:c.649dup	p.(Arg217Profs*8)	Heterozygous	Maternal	Benign familial infantile seizures 2	Autosomal Dominant	Pathogenic
061	Diagnostic	18p11.32p11.21	chr18:g.11358_15286250del	-	-	Heterozygous	De novo		Autosomal Dominant	Pathogenic
062	Non-diagnostic									
063	Non-diagnostic									

064	Non-diagnostic (Carrier)	*PMM2*	chr16:g.8905010G>A	NM_000303.2:c.422G>A	p.(Arg141His)	Heterozygous	Maternal	Congenital Disorder of Glycosyla-tion Type Ia	Autosomal Recessive	Pathogenic
065	Diagnostic	2q24.1q24.3	chr2:g.156436452_167759008dup	-	-	Heterozygous	Unknown		Autosomal Dominant	Pathogenic
066	Non-diagnostic (VUS)	*NSF*	chr17:g.44788448_44788450dupCAG	NM_006178.3:c.1590_1592dup	p.(Ser531dup)	Heterozygous	De novo	DEE 96	Autosomal Dominant	VUS
067	Diagnostic	*RRAS2*	chr11:g.14380349C>T	NM_012250.5:c.68G>A	p.(Gly23Asp)	Heterozygous	De novo	Noonan syndrome 12	Autosomal Dominant	Pathogenic
068	Non-diagnostic (Carrier)	7q11.21 (*KCTD7*)	chr7:g.66101799_66138433del	-	-	Heterozygous	Paternal	Progressive Myoclonic Epilepsy 3 With or Without Intracellular Inclusions	Autosomal Recessive	Pathogenic
069	Non-diagnostic (Carrier)	*PEX1*	chr7:g.92131224dupT	NM_000466.2:c.2396dup	p.(Ser800Glufs*34)	Heterozygous	Maternal	Peroxisome Biogenesis Disorder	Autosomal Recessive	Pathogenic
070	Non-diagnostic									
071	Non-diagnostic									
072	Non-diagnostic									
073	Diagnostic	16p11.2	chr16:g.29633154_30200019del	-	-	Heterozygous	Not maternal		Autosomal Dominant	Pathogenic
074	Diagnostic	*SCN1A*	chr2:g.166848918C>G	NM_001165963.2:c.4867G>C	p.(Glu1623Gln)	Heterozygous	Maternal	Generalized Epilepsy with Febrile Seizures Plus Type 2	Autosomal Dominant	Likely Pathogenic
075	Non-diagnostic									
076	Diagnostic	*TUBA1A*	chr12:g.49580449_49580452delinsATCTCTGAATGCT	NM_001270399.1:c.168_171delinsAGCATTCAGAGAT	p.(Gly57delinsAlaPheArgAsp)	Heterozygous	De novo	TUA1A-Related Disorder	Autosomal Dominant	Likely Pathogenic
077	Non-diagnostic									
078	Diagnostic	9pterq22.33	chr9:g.10001_101149236dup	-	-		Mosaic		Autosomal Dominant	Pathogenic

	Diagnostic	15q22.2qter	chr15:g.59963540_102521392dup	-	-		Mosaic		Autosomal Dominant	Pathogenic
079	Non-diagnostic (VUS)	*DMXL2*	chr15:g.51742450A>T	NM_001174116.3:c.8780T>A	p.(Ile2927Asn)	Homozygous	Maternal and Paternal	DEE 81	Autosomal Recessive	VUS
080	Non-diagnostic									
081	Non-diagnostic									
082	Diagnostic	*KCNQ2*	chr20:g.62038666delG	NM_172107.4:c.1955del	p.(Pro652Argfs*278)	Heterozygous	Maternal	Benign Familial Neonatal Seizures 1	Autosomal Dominant	Likely Pathogenic
083	Non-diagnostic									
084	Diagnostic	*PRRT2*	chr16:g.29825024C>T	NM_145239.3:c.649C>T	p.(Arg217*)	Heterozygous	Maternal	Benign Familial Infantile Seizures 2	Autosomal Dominant	Pathogenic
085	Non-diagnostic (Carrier)	*PIGG*	chr4:g.515631G>A	NM_001127178.3:c.1515G>A	p.(Trp505*)	Heterozygous	Paternal	Neurodevel-opmental Disorder With or Without Hypotonia, Seizures, and Cerebellar Atrophy	Autosomal Recessive	Likely Pathogenic
086	Diagnostic	*DEAF1*	chr11:g.686953G>T	NM_021008.4:c.709C>A	p.(Pro237Thr)	Heterozygous	De novo	Vulto-van Silfout-de Vries syndrome	Autosomal Dominant	Likely Pathogenic
087	Non-diagnostic									
088	Diagnostic	*KCNJ6*	chr21:g.39087225C>T	NM_002240.5:c.235G>A	p.(Asp79Asn)	Heterozygous	De novo	KCNJ6-Related Disorder	Autosomal Dominant	VUS (considered likely clinically diagnostic)
089	Non-diagnostic									
090	Non-diagnostic (VUS)	*COL4A2*	chr13:g.111102699G>A	NM_001846.4:c.1237G>A	p.(Gly413Arg)	Heterozygous	Maternal	Brain Small Vessel Disease 2	Autosomal Dominant	VUS
091	Diagnostic	16p11.2	chr16:g.29652060_30199344del	-	-		De novo		Autosomal Dominant	Pathogenic
092	Diagnostic	*SCN2A*	chr2:g.166164386A>G	NM_021007.3:c.415A>G	p.(Ile139Val)	Heterozygous	De novo mosaic	DEE 11	Autosomal Dominant	Likely Pathogenic

093	Diagnostic	*SCN8A*	chr12:g.52184203 A>G	NM_014191.4:c.4441A>G	p.(Met1481Val)	Heterozygous	Maternal	SCN8A-Related Disorder	Autosomal Dominant	Likely Pathogenic
094	Non-diagnostic									
095	Non-diagnostic									
096	Non-diagnostic									
097	Non-diagnostic									
098	Non-diagnostic									
099	Diagnostic	*SLC6A5*	chr11:g.20622967_20622968delAG	NM_004211.5:c.296_297del	p.(Glu99Glyfs*22)	Homozygous	Maternal and Paternal	Hyperek-plexia 3	Autosomal Recessive	Pathogenic
100	Diagnostic	*KCNQ3*	chr8:g.133186542 G>A	NM_004519.4:c.988C>T	p.(Arg330Cys)	Heterozygous	Paternal	Benign Familial Neonatal Seizures 2	Autosomal Dominant	Pathogenic

GS: genome sequencing, VUS: Variant of uncertain significance

Supplementary Table 6: Additional Genome Sequencing Results

ID	Additional GS Results	Gene symbol and/or cytoband	Variant (g. nomenclature; hg19)	Variant (c. nomenclature)	Variant (p. nomenclature)	Zygosity	Inheritance	Associated Disease	Mode of Inheritance	Classification
005	Secondary finding	*HFE*	chr6:g.26093141G>A	NM_000410.3:c.845G>A	p.(Cys282Tyr)	Homozygous	Maternal and Paternal	Hereditary Hemochromatosis	Autosomal Recessive	Pathogenic
008	Additional VUS	*SLC4A3*	chr2:g.220500615delT	NM_201574.2:c.2272+2del	p.(?)	Heterozygous	Paternal	None Currently Described	Unknown	VUS
010	Carrier	*PCK1*	chr20:g.56138646delG	NM_002591.3:c.824del	p.(Gly275Valfs*21)	Heterozygous	Paternal	Phosphoenolpyru-vate carboxykinase deficiency, cytosolic	Autosomal Recessive	Likely Pathogenic
012	Additional VUS	*SYNJ1*	chr21:g.34037261C>A	NM_003895.3:c.2383G>T	p.(Ala795Ser)	Heterozygous	Maternal	SYNJ1-Related Disorder	Autosomal Recessive	VUS
015	VUS	*TSC1*	chr9:g.135787693A>G	NM_000368.4:c.889T>C	p.(Tyr297His)	Heterozygous	Paternal	TSC1-Related Tuberous Sclerosis	Autosomal Dominant	VUS
016	VUS	*TTN*	chr2:g.179475831A>G	NM_001267550.1:c.51025T>C	p.(Ser17009Pro)	Heterozygous	De novo	TTN-Related Disorder	Autosomal Dominant/ Recessive	VUS
026	VUS	*HNRNPH1*	chr5:g.179045164G>A	NM_001257293.1:c.697C>T	p.(Arg233Cys)	Mosaic (16/46 reads)	De Novo	HNRNPH1-Related Disorder	Autosomal Dominant	VUS
		OPHN1	chrX:g.67433788C>G	NM_002547.2:c.513G>C	p.(Arg171Ser)	Hemizygous	Maternal	OPHN1-Related Disorder	X-linked	VUS
027	Carrier	*PMM2*	chr16:g.8905010G>A	NM_000303.2:c.422G>A	p.(Arg141His)	Heterozygous	Unknown (both parents carriers)	PMM2-Related Congenital Disorder of Glycosylation	Autosomal Recessive	Pathogenic
032	Carrier	*TRIT1*	chr1:g.40310290delA	NM_017646.4:c.1029del	p.(Val344Serfs*4)	Heterozygous	Paternal	TRIT1-Related Disorder	Autosomal Recessive	Likely Pathogenic
040	(Incidental) VUS	*FH*	chr1:g.241682896G>A	NM_000143.3:c.127C>T	p.(Arg43*)	Heterozygous	Maternal	Hereditary Leiomyomatosis and Renal Cell Cancer	Autosomal Dominant	VUS
065	VUS	*TRPM3*	chr9:g.73151536C>G	NM_020952.4:c.3998G>C	p.(Trp1333Ser)	Heterozygous	Unknown	DEE	Autosomal Dominant	VUS

079	Incidental Diagnostic Finding	*TYR*	chr11:g.88960984 T>A	NM_000372.5:c.1 037-7T>A	p.(?)	Homozygous	Maternal and Paternal	Oculocutaneous Albinism Type IA/IB	Autosomal Recessive	Pathogenic
084	Secondary finding	*HFE*	chr6:g.26093141G >A	NM_000410.3:c.8 45G>A	p.(Cys282Tyr)	Homozygous	Maternal and Paternal	Hemochromatosis Type 1	Autosomal Recessive	Pathogenic
085	Additional Carrier	*PLPBP*	chr8:g.37620217G >C	NM_007198.4:c.4 0G>C	p.(Gly14Arg)	Heterozygous	Paternal	Early-Onset Vitamin B6-Dependent Epilepsy	Autosomal Recessive	VUS
094	Incidental Diagnostic Finding	*F8*	chrX:g.154159916 G>A	NM_000132.4:c.2 149C>T	p.(Arg717Trp)	Hemizygous	Maternal	Hemophilia A	X-linked Recessive	Pathogenic
098	Incidental Diagnostic Finding	*SGMS2*	chr4:g.108817103 G>T	NM_001136257.2: c.394G>T	p.(Glu132*)	Heterozygous	Maternal	Calvarial Doughnut Lesions with Bone Fragility with or without Spondylometaphy seal Dysplasia	Autosomal Dominant	Likely Pathogenic

GS: genome sequencing, VUS: Variant of uncertain significance

Supplementary Table 7: Impact of Genome Sequencing Results

ID	Impact of Genome Sequencing
001	Influenced treatment (started vigabatrin, pursued epilepsy surgery), potential precision therapy (everolimus/mTOR inhibitors), informed prognosis (increased SUDEP risk), informed genetic/reproductive counseling (monitoring for maternal symptoms, testing for maternal family members)
002	New workup (referral to neuromuscular clinic), informed prognosis (risk of neuromuscular disorder in childhood)
005	New workup (Ophthalmology)
008	New workup (EKG)
010	New workup (referral to Oncology-Genetics)
011	New workup (referral to cardiology and ophthalmology, renal ultrasound), informed prognosis (risk of DD/ID)
014	New workup (referral to ophthalmology), informed prognosis (risk of DD/ID)
015	Potential precision therapy (everolimus/mTOR inhibitors), new workup (referral to PTEN clinic, Endocrinology, and TSC clinic, PTEN cancer specific screening (thyroid, colon, and renal)), informed prognosis (risk of various cancers, risk of ASD, risk of DD/ID)
016	(Patient passed away prior to genetic diagnoses), informed genetic/reproductive counseling (new diagnosis of maternal myotonic dystrophy and maternal referral to neuromuscular clinic, testing for maternal family members, 60-70% recurrence risk (due to preferential allele selection))
018	Influenced treatment and potential precision therapy (avoid sodium channel blockers and consider ASMs used in Dravet syndrome), informed prognosis (increased SUDEP risk, risk of difficult to treat seizures, risk of severe DD/ID)
021	Influenced treatment and potential precision therapy (avoid Keppra (wean off) and consider sodium channel blockers (presumed gain of function variant), informed prognosis (risk of paroxysmal kinesigenic dyskinesia), informed genetic/reproductive counseling (testing for sibling)
023	Informed genetic/reproductive counseling (testing for maternal family member with developmental delay)
026	New workup (referral to cardiology and echocardiogram), informed prognosis (risk of DD/ID)
027	New workup (referral to ophthalmology), informed prognosis (risk of DD/ID)
028	Influenced treatment and potential precision therapy (wean off phenobarbital), informed prognosis (likely self-limited epilepsy)
029	New workup (referral to audiology and ophthalmology, abdominal ultrasound, metabolic testing, labs including LFTs and immunoglobulins), avoided workup (sleep study and VCUG given goals of care in the setting of genetic diagnosis), informed prognosis and redirection of care, informed genetic/reproductive counseling (testing for maternal family members)
030	Influenced treatment (protein restriction and consider thiamine supplementation), informed prognosis (usually fatal by one year old)
032	Informed prognosis (risk of ASD, risk of DD/ID)
034	Influenced treatment (continue oxcarbazepine, wean off other ASMs), potential precision therapy (sodium channel blockers (presumed mild gain of function variant)), informed prognosis (likely self-limited epilepsy)
035	Influenced treatment (continue ASMs instead of weaning), new workup (referred to developmental multidisciplinary team), avoided workup (metabolic testing including LP), informed prognosis (risk of DD, risk of ongoing seizures)
036	Influenced treatment (stopped sodium channel blockers in favor of other ASMs), avoided workup (MRI)
037	New workup (referral to palliative care), avoided workup (MRI), informed prognosis and redirection of care
039	New workup (referral to genetics and developmental multidisciplinary team), avoided workup (MRI)
040	New workup (referral to genetics), informed genetic/reproductive counseling (maternal referral to genetics)
041	Influenced treatment (stopped biotin and pyridoxine), potential precision therapy (sodium channel blockers), new workup (referral to genetics), avoided workup (MRI), informed prognosis (likely good prognosis)
044	Influenced treatment (weaned medication)
045	New workup (referral to genetics and nerve conduction study), informed prognosis (risk of Charcot-Marie-Tooth disease)
046	Influenced treatment (stopped biotin and pyridoxine), potential precision therapy (sodium channel blockers), new workup (referral to genetics and renal ultrasound), avoided workup (MRI), informed prognosis (likelihood of better prognosis given mosaic)
050	New workup (referral to genetics and ophthalmology, ERG/VEP), informed prognosis (risk of DD/ID)
052	Influenced treatment (started ketogenic diet), new workup (referral to genetics and ketogenic diet clinic), informed prognosis (risk of DD, risk of sleep, gastrointestinal, and motor issues)
054	Influenced treatment (stopped biotin and pyridoxine), new workup (referral to genetics, neurodisability, ketogenic diet clinic, and developmental multidisciplinary team), informed prognosis (risk of DD/ID, risk of movement disorder)

056	Influenced treatment and potential precision therapy (use of sodium channel blockers specifically carbamazepine), new workup (referral to genetics), avoided workup (LP), informed prognosis (risk of DD/ID)
058	Influenced treatment and potential precision therapy (continue vigabatrin instead of stopping, pursued surgery), new workup (referral to cardiology, genetics, nephrology, and ophthalmology, echocardiogram and renal ultrasound), informed prognosis (risk of DD/ID, risk of non-nervous system involvement), informed genetic/reproductive counseling (referral for mother, testing for siblings and maternal family members)
060	Influenced treatment (switched from phenobarbital to oxcarbazepine), potential precision therapy (sodium channel blockers), informed prognosis (likely self-limited epilepsy and normal development, risk of paroxysmal kinesigenic dyskinesia or hemiplegic migraines), informed genetic/reproductive counseling (testing for maternal family members)
061	New workup (referral to cardiology and ophthalmology, echocardiogram), informed prognosis (risk of DD/ID)
065	Influenced treatment (continue ASM instead of weaning), potential precision therapy (sodium channel blockers), informed prognosis (risk of DD/ID)
067	New workup (referral to audiology, cardiology, genetics, and ophthalmology), informed genetic/reproductive counseling (testing for siblings)
073	Influenced treatment (continue current ASM), potential precision therapy (sodium channel blockers), new workup (referral to genetics), informed prognosis (likely age-limited epilepsy, risk of cognitive difficulties)
074	Influenced treatment (choice of ASMs, seizure management plans), potential precision therapy (avoid sodium channel blockers, preferred ASMs, clinical trial NCT0444229), new workup (referral to immunization clinic), informed prognosis (increased SUDEP risk, risk of DD/ID), informed genetic/reproductive counseling (testing for maternal family members)
077	New workup (MRI)
078	New workup (referral to cardiology, genetic, and nephrology, echocardiogram and renal ultrasound)
079	New workup (referral to genetics and ophthalmology), informed genetic/reproductive counseling (testing for siblings)
082	Influenced treatment and potential precision therapy (carbamazepine and discontinue pyridoxine), informed prognosis (likely self-limited epilepsy and good developmental outcome)
084	Influenced treatment and potential precision therapy (changed ASM to carbamazepine), new workup (referral to genetics), informed prognosis (likely self-limited epilepsy and normal development, risk of paroxysmal kinesigenic dyskinesia or hemiplegic migraines), informed genetic/reproductive counseling (etiology for maternal migraines, testing for siblings and maternal family members)
085	Redirection of care (negative results helped "rule out" treatable etiologies)
086	New workup (referral to Developmental Assessment of Genetically Susceptible Youth clinic, hearing test), informed prognosis (risk of DD/ID)
088	New workup (referral to genetics)
090	New workup (referral to genetics)
091	Influenced treatment and potential precision therapy (started carbamazepine), new workup (referral to Developmental Assessment of Genetically Susceptible Youth clinic and genetics, echocardiogram, hearing test, spine x-ray), informed prognosis (risk of DD/ID, growth issues, psychiatric illness, and cryptic congenital anomalies)
092	Influenced treatment and potential precision therapy (started carbamazepine), informed prognosis (risk of DD/ID)
093	Influenced treatment and potential precision therapy (oxcarbazepine), informed prognosis (risk of DD/ID), informed genetic/reproductive counseling (etiology for maternal history of seizures)
094	New workup (referral to hematology and hematology labs), informed genetic/reproductive counseling (maternal monitoring, testing for siblings)
098	New workup (referral to Genetics, ALP, x-rays), informed genetics/reproductive counseling (etiology of mother's history of hip fractures, maternal referral for bone mineral density scan and x-rays)
099	Influenced treatment and potential precision therapy (started clonazepam), new workup (referral to genetics), avoided workup (follow up EEGs), informed prognosis (likely improvement in apneas and stiffness, likely persistence of exaggerated startle response), informed genetic/reproductive counseling (testing for family members)
100	Influenced treatment and potential precision therapy (carbamazepine), informed prognosis (likely self-limited epilepsy and good developmental outcome), informed genetic/reproductive counseling (etiology for paternal history of seizures, testing for paternal family members)

ALP: alkaline phosphatase, ASD: autism spectrum disorder, ASM: anti-seizure medication, DD: developmental delay, EEG: electroencephalogram, EKG: electrocardiogram, ERG/VEP: electroretinogram/visual evoked potential, ID: intellectual disability, LP: lumbar puncture, LFTs: liver function tests, MRI: magnetic resonance imaging, SUDEP: sudden unexplained death in epilepsy, VCG: voiding cystourethrogram
